# Acquisition and clearance dynamics of *Campylobacter* spp. in children in low- and middle-income countries

**DOI:** 10.1016/j.epidem.2024.100749

**Published:** 2024-03

**Authors:** Dehao Chen, Arie H. Havelaar, James A. Platts-Mills, Yang Yang

**Affiliations:** a Department of Environmental and Global Health, College of Public Health and Health Professions, University of Florida, Gainesville, FL, USA; b Emerging Pathogens Institute, University of Florida, Gainesville, FL, USA; c Department of Epidemiology, Emory University, Atlanta, GA, USA; d Department of Animal Sciences, Institute of Food and Agricultural Sciences, University of Florida, Gainesville, FL, USA; e Global Food Systems Institute, University of Florida, Gainesville, FL, USA; f Division of Infectious Diseases and International Health, University of Virginia Health System, Charlottesville, VA, USA; g Department of Statistics, Franklin College of Arts and Sciences, University of Georgia, Athens, GA, USA

**Keywords:** *Campylobacter*, Acquisition, Clearance, Antibiotic effect, Markov model

## Abstract

The prevalence of *Campylobacter* infection is generally high among children in low- and middle-income countries (LMIC), but the dynamics of its acquisition and clearance are understudied. We aim to quantify this process among children under two years old in eight LMIC using a statistical modeling approach, leveraging enzyme-immunoassay-based *Campylobacter* genus data and quantitative-PCR*-*based *Campylobacter jejuni/coli* data from the MAL-ED study. We developed a Markov model to compare the dynamics of acquisition and clearance of *Campylobacter* across countries and to explore the effect of antibiotic usage on *Campylobacter* clearance. Clearance rates were generally higher than acquisition rates, but their magnitude and temporal pattern varied across countries. For *C. jejuni/coli*, clearance was faster than acquisition throughout the two years at all sites. For *Campylobacter* spp., the acquisition rate either exceeded or stayed very close to the clearance rate after the first half year in Bangladesh, Pakistan and Tanzania, leading to high prevalence. Bangladesh had the shortest (28 and 57 days) while Brazil had the longest (328 and 306 days) mean times from last clearance to acquisition for *Campylobacter* spp. and *C. jejuni/coli*, respectively. South Africa had the shortest (10 and 8 days) while Tanzania had the longest (53 and 41 days) mean times to clearance for *Campylobacter* spp. and *C. jejuni/col*, respectively. The use of Macrolide accelerated clearance of *C. jejuni/coli* in Bangladesh and Peru and of *Campylobacter* spp. in Bangladesh and Pakistan. Fluoroquinolone showed statistically meaningful effects only in Bangladesh but for both *Campylobacter* groups. Higher prevalence of *Campylobacter* infection was mainly driven by a high acquisition rate that was close to or surpassing the clearance rate. Acquisition rate usually peaked in 11–17 months of age, indicating the importance of targeting the first year of life for effective interventions to reduce exposures.

## Highlights

• Our study leverages longitudinal multi-site MAL-ED data at monthly scale.
• Clearance rates were generally higher than acquisition rates.
• Higher prevalence of *Campylobacter* was primarily driven by high acquisition rate.
• Acquisition rate usually peaked during 11 to 17 months of age.
• Macrolides accelerated clearance of *Campylobacter* in Bangladesh, Peru and Pakistan.

## 1 Introduction

Colonization by enteric pathogens, including *Campylobacter* spp., is a well-documented risk factor for malnutrition in children under five years of age (CU5) in low- and middle-income countries (LMIC) (Chen et al., 2021, Rogawski et al., 2018). The Global Enteric Multicenter Study (GEMS), a case-control study conducted in seven countries in Africa and Asia, found that *Campylobacter* was a leading pathogen responsible for diarrhea in CU5 (Liu et al., 2014, Liu et al., 2016). The Etiology, Risk Factors, and Interactions of Enteric Infections and Malnutrition and the Consequences for Child Health and Development (MAL-ED) project was a multi-national birth cohort study designed to evaluate the association of enteric pathogen infections with malnutrition and further health effects among children under two years of age in eight LMIC (Miller et al., 2014). This study has associated multiple household risk factors with *Campylobacter* infection (Amour et al., 2016). In addition, along with other recent single-site studies, MAL-ED associated (cumulative) infection of *Campylobacter* with impaired gut functions like gut inflammation and increased intestinal permeability (Amour et al., 2016, Kosek et al., 2017, Iqbal et al., 2019) and with undernutrition outcomes such as stunted growth and reduced weight gain (Rogawski et al., 2018, Amour et al., 2016, Lee et al., 2013).

Prevalence of *Campylobacter* usually increases in the early stage of life as infants transit to non-breastmilk food and more activities (Amour et al., 2016), but the exact time-varying patterns of the rate of acquisition (or equivalently, force of infection [FOI]) and the rate of clearance remain under-investigated, mainly due to the sparsity of appropriately designed longitudinal studies. Compared to prevalence, acquisition and clearance rates better capture the temporal changes at the exposure level and in immunity development. Over the past two decades, only two studies assessed the FOI of *Campylobacter* in children of heterogeneous age ranges in LMIC’s settings, and the FOI in both studies was estimated by seroconversion rate at an annual scale based on IgG data (Arnold et al., 2019, Arzika et al., 2022). Although the incidence proportions/rates of *Campylobacter* spp. in the MAL-ED population has been described elsewhere (Haque et al., 2019), those measures are the gross estimates of the whole follow-up. The clearance and acquisition process of *Campylobacter* in CU5 has only been descriptively illustrated in a study in Zanzibar based on a single two-week follow-up after diarrhea (Andersson et al., 2017). There are few studies on clearance/acquisition of subclinical infections, which are associated with growth (Rogawski et al., 2018). Less is known about risk/protective factors for acquisition and clearance in LMIC, e.g., the effect of antibiotics on clearance. Existing intervention studies aimed at improving linear growth by reducing enteric infections, using either water, sanitation, and hygiene measures (Pickering et al., 2019) or antibiotic treatments (DeBoer et al., 2021). However, none of these interventions showed appreciable effectiveness, possibly because of high FOI and/or long-lasting colonization. Understanding the dynamics of acquisition and clearance in young children could potentially inform the design and evaluation of future interventions. To date, MAL-ED remains the sole multisite study that garnered prospective longitudinal data on *Campylobacter* infection and antibiotic use in young children, offering a unique opportunity to quantify such dynamics (Rogawski et al., 2018). Here, we present a modeling analysis of the time-varying acquisition and clearance dynamics of *Campylobacter* spp. in the MAL-ED study using a simulation-validated two-stage discrete-time Markov process model. We also explored the effect of antibiotics on the time to clearance of *Campylobacter* spp.

## 2 Methods

### 2.1 Study design, field procedures, and populations

The study design of MAL-ED (enrollment scheme, stool sample collection, laboratory tests and surveillance of antibiotic use), along with the relevant epidemiological findings, has been presented elsewhere (Amour et al., 2016, Houpt et al., 2014, Miller et al., 2014, Rogawski et al., 2017, Rogawski et al., 2018). Briefly, between 2009 and 2014, routine non-diarrheal stool samples were collected monthly during 24 months of follow-up, in the following eight sites: Haydom, Tanzania; Venda, South Africa; Naushero Feroze, Pakistan; Dhaka, Bangladesh; Vellore, India; Bhaktapur, Nepal; Fortaleza, Brazil; and Loreto, Peru (Rogawski et al., 2018, Platts-Mills et al., 2018). During twice-weekly household visits, the reported history of antibiotic use was collected and validated using the packaging of the antibiotics or prescriptions from healthcare providers, and if a caregiver reported a diarrheal episode in a child, a diarrheal stool sample was garnered for testing (Amour et al., 2016, Rogawski et al., 2017). Diarrheal stool samples might substitute for routine non-diarrheal stool samples if the twice-weekly visit overlapped with a monthly visit. All samples during the 24-month follow-up were assayed by qPCR, using primers specific for *Campylobacter jejuni* and *Campylobacter coli* (Platts-Mills et al., 2018, Platts-Mills et al., 2014). Samples from months 1–12, 15, 18, 21, and 24 were also assayed by enzyme immunosorbent assay (EIA), which detected a broad group of *Campylobacter* spp. (consisting of *Campylobacter troglodytis*, Candidatus *Campylobacter infans*, *Campylobacter concisus*, *Campylobacter upsaliensis*, *C. jejuni*, and *C. coli*) (Platts-Mills et al., 2014, Platts-Mills et al., 2015). Hereafter, we refer to the qPCR- and EIA-detected *Campylobacter* groups as *C. jejuni/coli* and *Campylobacter* spp., respectively.

### 2.2 Statistical analysis

#### 2.2.1 Discrete-time two-state Markov model

We consider a two-state Markov model with transitions from the state of non-colonized to colonized by *Campylobacter* (acquisition) and vice versa (clearance) for each child. Let $X_{\mathrm{it}}$ indicate the colonization status (1 =colonized, 0 =not colonized) on day t during the follow-up of the $i^{\mathrm{th}}$ child, where t=0 and ${t=T}_{i}$ correspond to the days of birth and of the final follow-up of the child. We assumed this was a time-inhomogeneous process with the following single-day transition probability matrix:

$$P_{t}=\left(\begin{array}{cc} 1-p_{t} & p_{t}\\ q_{t} & 1-q_{t} \end{array}\right)$$

where $p_{t}\;$and$\;q_{t}\;$are respectively the daily acquisition probability (0→1) and clearance probability (1→0) for the transition from day t to t+1. Accordingly, $1-p_{t}$ and $1-q_{t}$ are respectively the probabilities of remaining in the non-colonized and colonized states. We use daily acquisition/clearance probability and acquisition/clearance rate interchangeably, as the two are very similar when a daily probability is small. To allow for temporal variation of the transition probabilities, we modeled logit-transformed $p_{t}\;$and$\;q_{t}\;$as quadratic polynomial functions of time via logit transformations in the following way:

$$u_{t}=\mathrm{logit}\left(\frac{p_{t}+q_{t}}{K}\right)=a+\mathrm{bt}+ct^{2}$$

$$v_{t}=\mathrm{logit}\left(\frac{p_{t}}{p_{t}+q_{t}}\right)=d+\mathrm{et}+ft^{2}$$

Here K is an upper bound to constrain the sum of $p_{t}\;$and$\;q_{t}$ (i.e., $p_{t}+q_{t}<K$) to ensure their values are biologically possible, which is assumed known. As the parameter spaces of $p_{t}+q_{t}\;$and $\frac{p_{t}}{p_{t}+q_{t}}$ are orthogonal, this parameterization improves identifiability of $p_{t}\;$and$q_{t}$ and the fundamental parameters a, b, c, d, e, and f. $p_{t}$ and $q_{t}$ can be expressed in terms of $u_{t}$ and $v_{t}$ via $p_{t}=K*\mathrm{expit}\left(u_{t}\right)*\mathrm{expit}\left(v_{t}\right)$ and $q_{t}=K*\mathrm{expit}\left(u_{t}\right)*\left(1-\mathrm{expit}\left(v_{t}\right)\right)$. We did not model $p_{t}$ and $q_{t}$ directly as polynomials because they are constrained between 0 and 1. A previous SIR-type model estimated the rate of transitioning from full to partial immunity after *Campylobacter* infections to be approximately 13/year, which translates to a daily rate of 13/365≈0.0356 (Swart et al., 2012 Mar). Under the assumption that the duration of full immunity is approximately equivalent to the duration of colonization, the magnitude of $p_{t}$ and $q_{t}$ should be comparable to $1-e^{-0.0356}\approx 0.035$. To be conservative, we set K=0.3 for the analysis but performed sensitivity analyses with 0.2 and 0.5, and results were not affected. In cases where the quadratic term is difficult to identify (possibly due to low overall/peak prevalence), we employed a linear function (appendix Section 2). For the *C. jejuni/coli* data from Brazil where the observed prevalence was zero in the first two months of life (Fig. 4), we started model fitting from month two to improve identifiability.

For each of the two *Campylobacter* groups, we estimated the site-specific and month-dependent mean time to acquisition, mean time to clearance (or mean duration of colonization), and FOI. The acquisition interval and duration of colonization (or time to clearance) are geometric distributions with $p_{t}$ and $q_{t}$ as their corresponding parameters (i.e., acquisition interval = $\frac{1}{p_{t}}$ and duration of colonization = $\frac{1}{q_{t}}$). The overall acquisition intervals and durations of colonization over the two-year follow-up were reported as the medians and geometric means of all $\frac{1}{p_{t}}$ and $\frac{1}{q_{t}}$ of days proximal to a whole month (i.e., t∈{0, 30, 60, …,720}). Based on the estimated daily $p_{t}$, the FOI during a given period from day $t_{1}$ to day $t_{2}$ is calculated as $\lambda =\sum_{t=t_{1}}^{t_{2}}\left[-\log\left(1-p_{t}\right)\right]$, where $\left[t_{1},t_{2}\right]$ was taken as [0, 365] for year 1, [366, 730] for year 2 and [0, 730] for the two years combined. The FOI is essentially the cumulative hazard rate.

#### 2.2.2 Maximum likelihood inference

The likelihood for time-inhomogeneous Markov process is complex. To simplify computation, we approximated the likelihood assuming the transition probabilities were relatively constant over the time interval between each pair of adjacent observed colonization states, where the constant value is the average of the daily transition probabilities evaluated at the beginning, median, and ending days of each interval (appendix Section 1). Before applying the model to the MAL-ED data, we validated the identifiability of fundamental parameters in a simulation study (appendix Section 2). In this study, a model is considered non-identifiable if (1) the fitted curve of $p_{t}+q_{t}$ is very close to its upper bound *K*; or (2) the estimated variance of any fundamental parameter (i.e, one of the regression coefficients *a, b, c, d, e, f*) is much larger than those of the remaining fundamental parameters. All results are based on identifiable models. When multiple models are identifiable, we further use the Bayesian Information Criterion (BIC) and the determinant of the Hessian matrix to help with model selection, the latter being related to D-optimality in experimental design theory (Emery and Nenarokomov, 1998). Please refer to appendix Section 2 for a thorough discussion on model identifiability and selection. The goodness-of-fit of each site-specific model was assessed by comparing the model-simulated trajectory of the prevalence to the observed counterpart (appendix Section 3).

#### 2.2.3 Characterizing effects of recent antibiotic use on clearance

The clearance probability at a given day was adjusted for the use of antibiotics during an eight-day window (and a 16-day window as a sensitivity analysis) preceding and including that day by using a complementary log-log regression, and the antibiotic effect is represented by a relative log time to clearance (RLTC). The RLTC is analogous to relative risk, because the time to clearance affected by antibiotics is expectedly shorter, associated with a smaller risk of colonization-induced health consequences (appendix Section 4).

## 3 Results

**Fig. 1:**
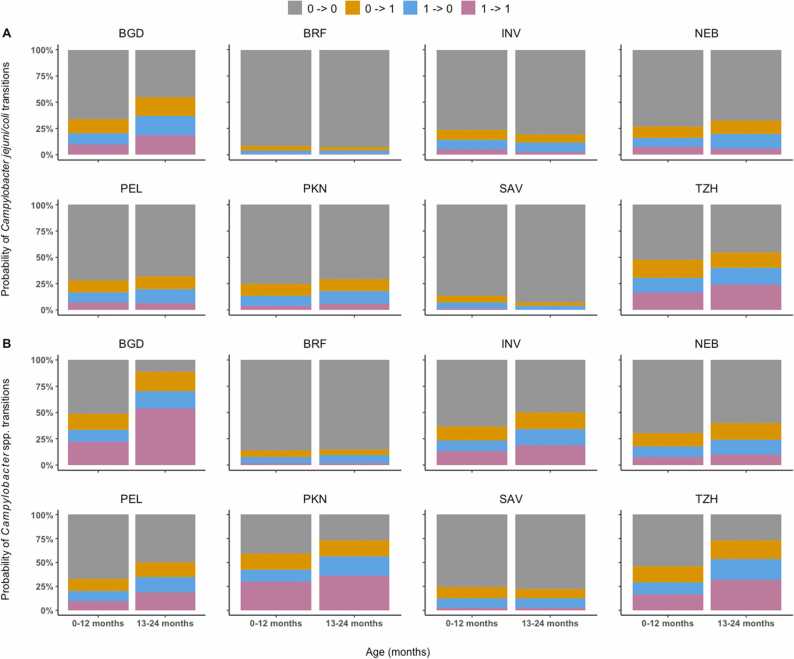
Site-specific relative frequencies of observed transitions of *Campylobacter jejuni/coli* (A) and *Campylobacter* spp. (B) between uncolonized and colonized states during 0–12 months (left column) and 13–24 months (right column) of age. Study sites include: Dhaka, Bangladesh (BGD); Vallore, India (INV); Bhaktapur, Nepal (NEB); Naushero Feroze, Pakistan (PKN); Venda, South Africa (SAV); Haydom, Tanzania (TZH); Fortaleza, Brazil (BRF); Loreto, Peru (PEL).

The number of children who had ≥ 1 stool sample assayed for *Campylobacter* spp. or *C. jejuni/coli* were 210, 165, 227, 227, 194, 246, 237, and 209 for the sites in Bangladesh, Brazil, India, Nepal, Peru, Pakistan, South Africa, and Tanzania, respectively. The observed frequencies of the four transition types differed substantially across sites and age (Fig. 1). We referred to transitions involving the colonization state (0→1, 1→0 and 1→1) as colonization activities. In the first year of life, relative frequencies of colonization activities of *C. jejuni/coli* were already high in Bangladesh and Tanzania, relatively low in Brazil and South Africa, and intermediate in the other four countries (Fig. 1A). The colonization activities of *C. jejuni/coli* were increasing from year 1 to year 2 of age in five of the eight countries but the magnitude of increase was more notable again in Bangladesh and Tanzania. The colonization activities were flat in Brazil and dwindled in India and South Africa. In the second year of age, colonization activities in Bangladesh and Tanzania were characterized by excessive sustained colonization or reinfection, indicated by a large proportion of 1→1. As expected, the levels of colonization activities of *Campylobacter* spp. in each country and year were higher than those of *Campylobacter jejuni/coli* (Fig. 1B). In year 1, Bangladesh, Pakistan and Tanzania had the leading levels of *Campylobacter* spp. colonization activities, followed by India, Nepal, and Peru. These six countries also observed notable increases in the activities from year 1 to year 2, whereas Brazil and South Africa showed no obvious changes over the two years. High levels of 1→1 of *Campylobacter* spp. in the second year were seen in Bangladesh, Pakistan, and Tanzania.

**Fig. 2:**
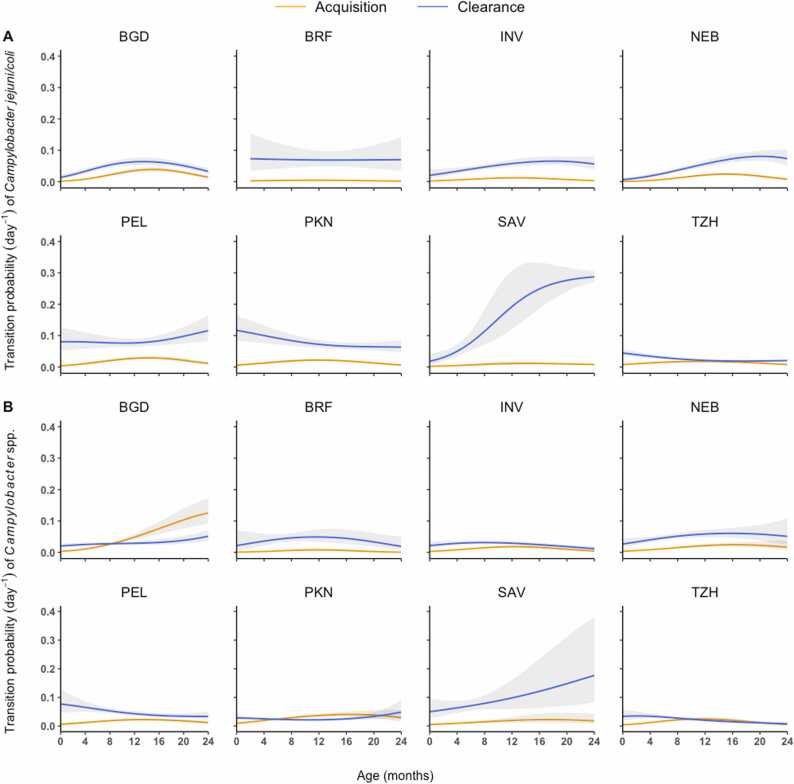
Model-estimated site-specific daily acquisition (orange) and clearance (blue) probabilities of *Campylobacter jejuni/coli* (A) and *Campylobacter* spp. (B). 95% asymptotic confidence intervals are shown as gray error bands. These estimates are not adjusted for antibiotic use. Study sites include: Dhaka, Bangladesh (BGD); Vallore, India (INV); Bhaktapur, Nepal (NEB); Naushero Feroze, Pakistan (PKN); Venda, South Africa (SAV); Haydom, Tanzania (TZH); Fortaleza, Brazil (BRF); Loreto, Peru (PEL).

Based on the model-estimated coefficients (appendix Tables S2 and S3), we plotted the model-estimated temporal patterns of daily acquisition and clearance probabilities of *C. jejuni/coli* and *Campylobacter* spp. in Fig. 2, which varied considerably across sites with few commonalities. The acquisition probability peaked between months 11 and 17 at most sites for both *Campylobacter* groups with few exceptions. For C. *jejuni/coli*, clearance probabilities were noticeably higher than acquisition probabilities at nearly all sites and all time points except for months 12–16 in Tanzania, when the two probabilities were comparable (Fig. 2A). For *Campylobacter* spp., the two probabilities were closer to each other as compared to those for *C. jejuni/coli*, and crossover of the two probabilities occurred (Fig. 2B). In Bangladesh, the acquisition probability exceeded the clearance probability around month nine and kept increasing afterwards. In Pakistan and Tanzania, the acquisition probability surpassed the clearance probability slightly towards the end of year 1 but stayed close during year 2. Acquisition and clearance probabilities for *Campylobacter* spp. were also close during year 2 in India and Peru. In particular, the increase in the clearance probability was steady and relatively steep during the whole two years in South Africa for both *C. jejuni/coli* and *Campylobacter* spp. These model-based results are in line with the summary data in Fig. 1 and seem to imply that a high level of the colonization activities (i.e., transitions of 0→1, 1→0 and 1→1) is associated with two conditions: (1) the acquisition probabilities are relatively high, and (2) the clearance probabilities are lower than or close to the acquisition probabilities. Examples are Bangladesh, Pakistan, India, and Tanzania in Fig. 1B and Fig. 2B.

We obtained the mean times to acquisition and to clearance as the reciprocals of the daily acquisition and clearance probabilities respectively (appendix Fig. S2). As the probabilities are age-dependent, these duration estimates are also age-dependent. In most countries and for both *Campylobacter* groups, the time to acquisition decreased during year 1 but increased during year 2 with the lowest value near the end of year 1. There is no consistent temporal pattern for the time to clearance. To aid interpretation and comparison, we summarized the real-time daily estimates by taking their median and average over the months (appendix Table S5). Bangladesh and Brazil respectively had the shortest (28 and 57 days) and the longest (328 and 306 days) mean times to acquisition for both *Campylobacter* spp. and *C. jejuni/coli*; and South Africa and Tanzania had the shortest (10 and 8 days) and the longest (53 and 41 days) mean times to clearance. The mean time to clearance of *C. jejuni/coli* was shorter than that of *Campylobacter* spp. in all countries except for Nepal (appendix Table S5).

Bangladesh had the highest two-year forces of infection (FOI), reaching 17.39 (95% CI: 14.82, 19.96) infection episodes/24 month for *C. jejuni/coli* and 42.36 (95% CI: 31.25, 53.47) for *Campylobacter* spp., followed by Peru for *C. jejuni/coli* (FOI=14.30, 95% CI: 11.90, 16.71) and Pakistan for *Campylobacter* spp. (FOI=22.79, 95% CI: 18.29, 27.29), whereas Brazil had the lowest FOI for both *Campylobacter* groups (appendix Fig. S3). The FOIs of the second year were larger than those of the first year at most sites. As expected, *Campylobacter* spp. had a larger two-year FOI than *C. jejuni/coli* in all countries except for Peru.

MAL-ED surveyed the usages of six antibiotic classes (penicillins, sulfonamides, macrolides, metronidazole, cephalosporins, and fluoroquinolones) (Rogawski et al., 2017). We excluded the classes that are known to be not active against *Campylobacter* from our analyses (Fitzgerald et al., 2008), and macrolides and fluoroquinolones were considered the classes of primary interest.

**Fig. 3:**
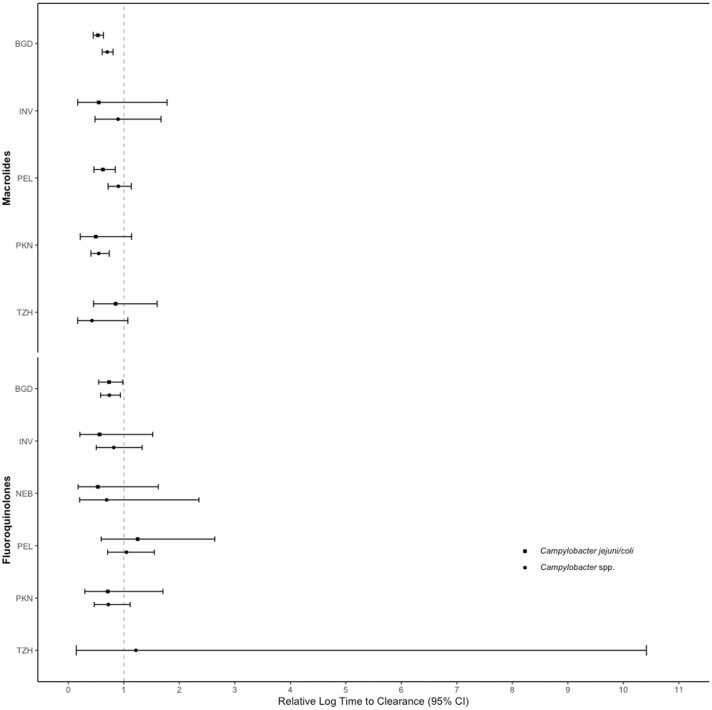
Site-specific effects of the use of macrolides and fluoroquinolones during one week prior to specimen collection on the clearances of *Campylobacter jejuni/coli* and *Campylobacter* spp. Point estimates and 95% confidence intervals of the relative log time to clearance (RLTC) were shown, which is defined as the ratio of the log time to clearance with antibiotic use to the log time to clearance without. Study sites include: Dhaka, Bangladesh (BGD); Vallore, India (INV); Bhaktapur, Nepal (NEB); Naushero Feroze, Pakistan (PKN); Venda, South Africa (SAV); Haydom, Tanzania (TZH); Fortaleza, Brazil (BRF); Loreto, Peru (PEL).

The use of macrolides or fluoroquinolones up to a week before a colonization episode was significantly associated with shortened durations of colonization for both *C. jejuni/coli* and *Campylobacter* spp. in Bangladesh (Fig. 3). A smaller relative log time to clearance (RLTC) indicates a stronger antibiotic effect on shortening the time to clearance (or equivalently, accelerating clearance). The RLTCs of *C. jejuni/coli* and *Campylobacter* spp. in Bangladesh were estimated as 0.53 (95% CI: 0.45, 0.63) and 0.70 (95% CI: 0.61, 0.81) for macrolides and 0.73 (95% CI: 0.55, 0.98) and 0.74 (0.58, 0.94) for fluoroquinolones. The use of macrolides was also associated with accelerated clearance of *C. jejuni/coli* in Peru (RLTC = 0.62, 95% CI: 0.46, 0.85) and *Campylobacter* spp. in Pakistan (RLTC = 0.55, 95% CI: 0.41, 0.74) (Fig. 3). At most sites, the RLTCs of macrolides were smaller for *C. jejuni/coli* than for *Campylobacter* spp., and both *Campylobacter* groups appeared to be more sensitive to macrolides than to fluoroquinolones (Fig. 3). For both antibiotic classes, most point estimates of RLTC were less than one, suggesting that they did in general accelerate clearance. A sensitivity analysis examining antibiotic use during a 16-day window preceding and including each colonization day yielded similar results (appendix Section 4 and Fig. S4). Antibiotic effects at some sites were either not estimated or estimated with wide confidence intervals due to rare antibiotic use (appendix Section 4). Hence, our ability to assess statistical significance varied across sites. To facilitate the interpretation of RLTC, we translated each statistically significant RLTC value to a ratio of mean times (instead of log mean times) to clearance with vs. without antibiotic use. The calculation of this ratio depends on the baseline daily clearance probability in the absence of antibiotics, for which we used the average baseline daily clearance probability throughout the two-year study period (appendix Fig. S5). For example, the ratios of mean times to clearance for macrolides were about 0.20 for *Campylobacter* spp. in Pakistan and 0.24 for *C. jejuni/coli* in Bangladesh.

**Fig. 4:**
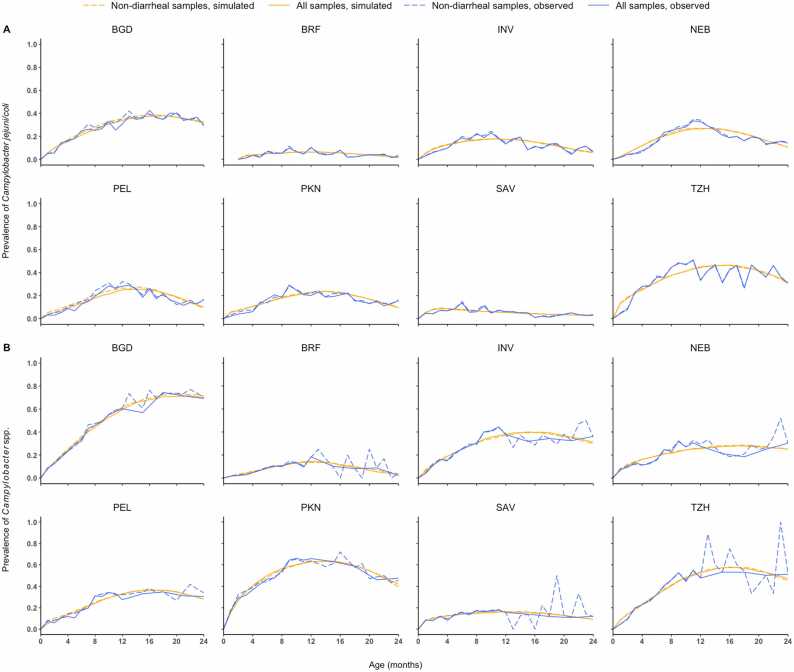
Site-specific observed and simulated longitudinal trends of prevalence of *Campylobacter jejuni/coli* (A) and *Campylobacter* spp. (B) among all and non-diarrheal samples. The simulated longitudinal trends were averaged from 100 realizations generated using fundamental parameters estimated from the corresponding observed data.

The model-simulated epidemic trajectories of *C. jejuni/coli* and *Campylobacter* spp. were highly consistent with the observed ones at all sites regardless of whether all samples or non-diarrheal samples were modeled, indicating adequate goodness-of-fit (Fig. 4). Overall, the prevalence of *C. jejuni/coli* and *Campylobacter* spp. increased sharply in the first year of age, with different peak values and peaking times at different sites, and then plateaued or declined gradually during the second year. As clearance of *C. jejuni/coli* was faster than *Campylobacter* spp. at all sites except Nepal (Fig. 2, appendix Table S5), the peak prevalence of *C. jejuni/coli* was generally lower than that of *Campylobacter* spp. (Fig. 4). At sites in Bangladesh, Pakistan and Tanzania where acquisition and clearance rates of *Campylobacter* spp. crossed over (Fig. 2), the overall and peak prevalence rates of *Campylobacter* spp. were higher than those at the other sites (Fig. 4). In Bangladesh where acquisition of *Campylobacter* spp. was faster than clearance in the second year (Fig. 2), the peak of prevalence was delayed to the end of the second year (Fig. 4).

## 4 Discussion

Harnessing the longitudinal data from MAL-ED, we found that a high burden of *Campylobacter* in young children in LMICs was primarily driven by a relatively high acquisition rate that was close to or exceeding the clearance rate. We also found the acquisition rate was rising during the first year and peaked between months 11 and 17 at most sites for both *C.jejuni/coli* and *Campylobacter* spp. The temporal pattern of clearance rates, on the other hand, varied substantially from country to county. The antibiotic class of macrolides tended to be more active on *C. jejuni/coli* than on *Campylobacter* spp., and both *Campylobacter* groups seem to be more sensitive to macrolides than to fluoroquinolones.

While the epidemiology of the thermotolerant *C. jejuni/coli* has been well studied given their health impact in the developed world (Friedman, 2000), little is known about the other, mainly the non-thermotolerant species in the *Campylobacter* genus. In the first two years of life, while the FOI of *Campylobacter* spp. was higher than *C. jejuni/coli* (except for Peru), clearance of *C. jejuni/coli* was often faster than of *Campylobacter* spp. Care should be taken when interpreting the comparisons between *C. jejuni/coli* and *Campylobacter* spp., as the former is a subset of the latter. If co-colonization of multiple *Campylobacter* species is common, then our results do not suggest higher FOI or faster clearance of *C. jejuni/coli* compared to other *Campylobacter* species, as it is expected that colonization with any species is faster and clearance of all species is slower when compared to a specific species. On the other hand, if species-level data (or group-level data such as thermotolerant and non-thermotolerant) were available, we could evaluate if they competed for ecological niche, and if they did not co-colonize often, then our estimates for *Campylobacter* spp. could be interpreted as an average between *C. jejuni/coli* and other *Campylobacter* species. The reversed order in FOI between *C. jejuni/coli* and *Campylobacter* spp. in Peru is possible if *C. jejuni/coli* were the dominant species among *Campylobacter* spp., as then the presumably higher sensitivity of qPCR in comparison to EIA would lead to a higher detection frequency.

Driven by environmental exposures, human hosts acquire *Campylobacter* through multiple pathogenesis factors (e.g., motility, chemotaxis, adhesion) (Van Vliet and Ketley), triggering innate, humoral, and cell-mediated immune responses in human hosts (Janssen et al., 2008). While recurrent exposures to *Campylobacter* trigger short-term protective effects against symptoms (e.g., diarrhea), it is unclear whether these protective effects accelerate clearance (Janssen et al., 2008). As reflected in our findings, the daily acquisition probability was temporally variant and peaked between 11–17 months at most sites. Future interventions may target the first year of life when acquisition is accelerating. Bangladesh is the only country with prolonged growth of the acquisition rate of *Campylobacter* spp. throughout the first two years of life, likely implying that more efforts are needed to reduce *Campylobacter* burden in children there. In contrast to acquisition, the trend of the clearance probability of *Campylobacter* appears to be more complicated, increasing with age at some sites but flat or even slightly decreasing at others, possibly related to country differences in maternally acquired immunity in early months, development of human immune system, antibiotics use, and nutrition status (Simon et al., 2015, Sahin et al., 2003). For example, the steady increase of clearance probability of both *C. jejuni/coli* and *Campylobacter* spp. in South Africa to levels not observed in any other country may explain the lower prevalence and less frequent diarrhea and use of antibiotics (Rogawski et al., 2017). The different clearance probabilities across countries might be related to different levels of the immunosuppressive effects associated with stunting. Slow clearance might also be related to environmental enteric dysfunction (a key pathway to stunting) that increases the translocation of pathogen-associated molecular products such as lipopolysaccharide and thereby weakens host immunity (Gwela et al., 2019). The complex interactions between environmental exposure, immune response, poor gut health, and undernutrition might have amplified the “vicious cycle of diseases of poverty” (Guerrant et al., 2013).

Our finding on the stronger antibiotic effects on the clearance of *C. jejuni/coli* than *Campylobacter* spp. may imply the non-thermotolerant group was less sensitive to these two antibiotic classes than other species in the genus. Our finding on the larger effect of macrolides on clearance was corroborated by the fact that resistance to fluoroquinolones in *Campylobacter* is more common (Sproston et al., 2018), making macrolides the empirical medication of choice for campylobacteriosis (Bolinger and Kathariou, 2017, Engberg et al., 2001), though emerging resistance to macrolides was also documented (Schiaffino et al., 2019, Mukherjee et al., 2014). A randomized controlled trial (RCT) conducted in one MAL-ED site, Haydom in Tanzania (TZH), found azithromycin (a macrolide antibiotic) treatment did not improve linear growth in children under 18 months of age (DeBoer et al., 2021). Although children were not tested for *Campylobacter* spp. in that trial, our finding of the longest mean time to clearance at TZH among all MAL-ED sites, alongside the non-significant effects of macrolides on the clearance of *Campylobacter* spp. at TZH, might indicate that the failure of this RCT to improve growth was likely due to slow or insufficient clearance of enteric infections.

Our findings are grounded on the novel Markov model validated by a simulation study. Markov models have been applied in studies of acquisition/clearance in a variety of pathogens such as *Neisseria meningitidis* in African countries (MenAfriCar Consortium, 2016), antibiotic-resistant *Staphylococcus aureus* in a nosocomial setting in the United Kingdom (UK) (Verykouki et al., 2016), and *Streptococcus pneumoniae* in UK households (Melegaro et al., 2007). Despite the longitudinal nature of these studies, many of them modeled acquisition and clearance as time-invariant, an untenable assumption considering the complex biological interactions among pathogens, hosts, and environment. In contrast, our model captures time-varying transition dynamics with a flexible parametric structure while ensuring model identifiability. This framework can be extended to nonparametric structures for more flexibility, e.g., using splines in the regression of the acquisition and clearance probabilities, if supported by abundant data.

A limitation of this modeling study is that the sensitivity and specificity of EIA for *Campylobacter* spp. and qPCR for *C. jejuni/coli* were assumed perfect, and misclassification of the colonization status is possible. Another important limitation is that MAL-ED is an observational study, and our findings on the effect of antibiotics on *Campylobacter* clearance should be interpreted with caution. As there was only one site in each country, our results might not be generalizable to the country level. In addition, the current modeling framework does not distinguish between different lineages, especially for the *Campylobacter* spp. group. The higher prevalence of EIA-detected than qPCR-detected infections suggests that some other (non-thermotolerant) species co-occur in addition to *C. jejuni/coli* and that the reservoirs and transmission pathways of these species might be different, resulting in more heterogeneity. The estimated acquisition and clearance rates should be interpreted as the average at the genus or group level and cannot be generalized to specific *Campylobacter* species. This model can be further refined to achieve improved identifiability and to yield species-level results if data on source of contact and genotype become available. Finally, while our model provides a flexible framework, the identifiability of the model parameters depends on a variety of factors including but not limited to the number of samples at each time point, sampling frequency, and the choice of the upper bound K. In practice, we recommend fixing *K* at a value based on empirical knowledge and performing sensitivity analyses. Although our results appeared insensitive to moderate changes in K, the sensitivity may further depend on sample size and sampling frequency, which warrants a systematic investigation in the future.

## 5 Conclusion

In summary, this work uncovered the acquisition and clearance dynamics of *Campylobacter* spp. in children in low-resource settings and the effect of antibiotics on their clearance. Future studies may consider collecting more data on host and environmental characteristics to better explain the heterogeneity in acquisition and clearance patterns across countries. Whenever resources permit, a complete profile of *Campylobacter* at the species level coupled with a multi-species modeling framework would reveal more insights into the acquisition and clearance dynamics of different species and their joint impact on children’s gut health and growth, which will inform future risk assessments and intervention strategies.

## Ethical approval statement

This manuscript is a secondary analysis of deidentified data from the MAL-ED study. All MAL-ED study sites received ethical approval from their respective governmental, local institutional, and collaborating institutional review boards. Written informed consent was obtained from the parent or guardian of each child. (see Amour et al., 2016, https://doi.org/10.1093/cid/ciw542).

## Authors contributions

DC and JAP-M have verified the underlying data. All authors had full access to all the data in the study and accepted the responsibility to submit the manuscript for publication. The Markov model was conceived by YY, with inputs from DC and AHH. DC led data analysis and manuscript drafting. All authors contributed to the interpretation of the results and participated in editorial revisions.

## Funding

DC, AH, YY were funded by the 10.13039/100000200United States Agency for International Development (USAID) Bureau for Food Security under Agreement #AID-OAA-L-15–00003 as part of Feed the Future Innovation Lab for Livestock Systems, and by the 10.13039/100000865Bill & Melinda Gates Foundation OPP#1175487. JAP-M was supported by the 10.13039/100000002National Institutes of Health (grant number R01AI153254).

## Declaration of Competing Interest

None.

## Data Availability

Data used in this study can requested from the Clinical Epidemiology Database REsources (http://ClinEpiDB.org).

## References

[bib1] Amour C., Gratz J., Mduma E.R., Svensen E., Rogawski E.T., McGrath M. (2016). Epidemiology and impact of campylobacter infection in children in 8 low-resource settings: results from the MAL-ED study. Clin. Infect. Dis..

[bib2] Andersson M.E., Elfving K., Shakely D., Nilsson S., Msellem M., Trollfors B. (2017). Rapid clearance and frequent reinfection with enteric pathogens among children with acute diarrhea in Zanzibar. Clin. Infect. Dis..

[bib3] Arnold B.F., Martin D.L., Juma J., Mkocha H., Ochieng J.B., Cooley G.M. (2019). Enteropathogen antibody dynamics and force of infection among children in low-resource settings. Elife.

[bib4] Arzika A.M., Maliki R., Goodhew E.B., Rogier E., Priest J.W., Lebas E. (2022). Effect of biannual azithromycin distribution on antibody responses to malaria, bacterial, and protozoan pathogens in Niger. Nat. Commun..

[bib5] Bolinger H., Kathariou S. (2017). The current state of macrolide resistance in Campylobacter spp.: trends and impacts of resistance mechanisms. Appl. Environ. Microbiol.

[bib6] Chen D., Mechlowitz K., Li X., Havelaar A.H., McKune S.L. (2021). Benefits and risks of smallholder livestock production on child nutrition in low- and middle-income countries. Front Nutr..

[bib7] DeBoer M.D., Platts-Mills J.A., Elwood S.E., Scharf R.J., McDermid J.M., Wanjuhi A.W. (2021). Effect of scheduled antimicrobial and nicotinamide treatment on linear growth in children in rural Tanzania: a factorial randomized, double-blind, placebo-controlled trial. PLoS Med.

[bib8] Emery A.F., Nenarokomov A.V. (1998). Optimal experiment design. Meas. Sci. Technol. [Internet].

[bib9] Engberg J., Aarestrup F.M., Taylor D.E., Gerner-Smidt P., Nachamkin I. (2001). Quinolone and macrolide resistance in Campylobacter jejuni and C. coli: resistance mechanisms and trends in human isolates. Emerg. Infect. Dis..

[bib10] Fitzgerald C., Whichard J., Nachamkin I. (2008). Campylobacter Species. Campylobacter.

[bib11] Friedman C.R. (2000). Epidemiology of Campylobacter jejuni infections on the United States and other industrialized nations. Campylobacter.

[bib12] Guerrant R.L., DeBoer M.D., Moore S.R., Scharf R.J., Lima A.A. (2013). The impoverished gut--a triple burden of diarrhoea, stunting and chronic disease.. Nat. Rev. Gastroenterol. Hepatol..

[bib13] Gwela A., Mupere E., Berkley J.A., Lancioni C. (2019). Undernutrition, host immunity and vulnerability to infection among young children. Pedia Infect. Dis. J..

[bib14] Haque M.A., Platts-Mills J.A., Mduma E., Bodhidatta L., Bessong P., Shakoor S. (2019). Determinants of Campylobacter infection and association with growth and enteric inflammation in children under 2 years of age in low-resource settings. Sci. Rep..

[bib15] Houpt E., Gratz J., Kosek M., Zaidi A.K.M., Qureshi S., Kang G. (2014). Microbiologic methods utilized in the MAL-ED cohort study. Clin. Infect. Dis..

[bib16] Iqbal N.T., Syed S., Kabir F., Jamil Z., Akhund T., Qureshi S. (2019). Pathobiome driven gut inflammation in pakistani children with environmental enteric dysfunction. PLoS One.

[bib17] Janssen R., Krogfelt K.A., Cawthraw S.A., Van Pelt W., Wagenaar J.A., Owen R.J. (2008). Host-pathogen interactions in Campylobacter infections: the host perspective. Clin. Microbiol Rev..

[bib18] Kosek M.N., Ahmed T., Bhutta Z.A., Caulfield L., Guerrant R.L., Houpt E., et al. Causal Pathways from Enteropathogens to Environmental Enteropathy: Findings from the MAL-ED Birth Cohort Study. EBioMedicine. 2017; 10.1016/j.ebiom.2017.02.024 PMC5405169 28396264

[bib19] Lee G., Pan W., Peñataro Yori P., Paredes Olortegui M., Tilley D., Gregory M. (2013). Symptomatic and asymptomatic campylobacter infections associated with reduced growth in peruvian children. PLoS Negl. Trop. Dis..

[bib20] Liu J., Kabir F., Manneh J., Lertsethtakarn P., Begum S., Gratz J. (2014). Development and assessment of molecular diagnostic tests for 15 enteropathogens causing childhood diarrhoea: a multicentre study. Lancet Infect. Dis..

[bib21] Liu J., Platts-Mills J.A., Juma J., Kabir F., Nkeze J., Okoi C. (2016). Use of quantitative molecular diagnostic methods to identify causes of diarrhoea in children: a reanalysis of the GEMS case-control study. Lancet.

[bib22] Melegaro A., Choi Y., Pebody R., Gay N. (2007). Pneumococcal Carriage in United Kingdom Families: estimating serotype-specific transmission parameters from longitudinal data. Am. J. Epidemiol..

[bib23] MenAfriCar Consortium (2016). Household transmission of Neisseria meningitidis in the African meningitis belt: a longitudinal cohort study. Lancet Glob. Health.

[bib24] Miller M., Acosta A.M., Chavez C.B., Flores J.T., Olotegui M.P., Pinedo S.R. (2014). The MAL-ED study: a multinational and multidisciplinary approach to understand the relationship between enteric pathogens, malnutrition, gut physiology, physical growth, cognitive development, and immune responses in infants and children up to 2 years of age in resource-poor environments. Clin. Infect. Dis..

[bib25] Mukherjee P., Ramamurthy T., Mitra U., Mukhopadhyay A.K. (2014). Emergence of high-level azithromycin resistance in Campylobacter jejuni isolates from pediatric diarrhea patients in Kolkata, India. Antimicrob. Agents Chemother..

[bib26] Pickering A.J., Null C., Winch P.J., Mangwadu G., Arnold B.F., Prendergast A.J. (2019). The WASH benefits and SHINE trials: interpretation of WASH intervention effects on linear growth and diarrhoea. Lancet Glob. Health.

[bib27] Platts-Mills J.A., Liu J., Gratz J., Mduma E., Amour C., Swai N. (2014). Detection of Campylobacter in stool and determination of significance by culture, enzyme immunoassay, and PCR in developing countries. J. Clin. Microbiol..

[bib28] Platts-Mills J.A., Babji S., Bodhidatta L., Gratz J., Haque R., Havt A. (2015). Pathogen-specific burdens of community diarrhoea in developing countries: a multisite birth cohort study (MAL-ED). Lancet Glob. Health.

[bib29] Platts-Mills J.A., Liu J., Rogawski E.T., Kabir F., Lertsethtakarn P., Siguas M. (2018). Use of quantitative molecular diagnostic methods to assess the aetiology, burden, and clinical characteristics of diarrhoea in children in low-resource settings: a reanalysis of the MAL-ED cohort study. Lancet Glob. Health.

[bib30] Rogawski E.T., Platts-Mills J.A., Seidman J.C., John S., Mahfuz M., Ulak M. (2017). Use of antibiotics in children younger than two years in eight countries: a prospective cohort study. Bull. World Health Organ.

[bib31] Rogawski E.T., Liu J., Platts-Mills J.A., Kabir F., Lertsethtakarn P., Siguas M. (2018). Use of quantitative molecular diagnostic methods to investigate the effect of enteropathogen infections on linear growth in children in low-resource settings: longitudinal analysis of results from the MAL-ED cohort study. Lancet Glob. Health.

[bib32] Sahin O., Luo N., Huang S., Zhang Q. (2003). Effect of Campylobacter-specific maternal antibodies on Campylobacter jejuni colonization in young chickens. Appl. Environ. Microbiol..

[bib33] Schiaffino F., Colston J.M., Paredes-Olortegui M., François R., Pisanic N., Burga R. (2019). Antibiotic resistance of Campylobacter species in a pediatric cohort study. Antimicrob. Agents Chemother..

[bib34] Simon A.K., Hollander G.A., McMichael A. (2015). Evolution of the immune system in humans from infancy to old age. Proc. R. Soc. B Biol. Sci..

[bib35] Sproston E.L., Wimalarathna H.M.L., Sheppard S.K. (2018). Trends in fluoroquinolone resistance in Campylobacter. Micro Genom..

[bib36] Swart A.N., Tomasi M., Kretzschmar M., Havelaar A.H., Diekmann O. (2012). The protective effects of temporary immunity under imposed infection pressure. Epidemics.

[bib37] Van Vliet A.H.M., Ketley J.M. Pathogenesis of enteric Campylobacter infection. 10.1046/j.1365-2672.2001.01353.x 11422560

[bib38] Verykouki E., Kypraios T., O’Neill P.D. (2016). Modelling the effect of antimicrobial treatment on carriage of hospital pathogens with application to MRSA. Biostatistics.

