# Supplementary material for: Acquisition and clearance dynamics of Campylobacter spp. in children in low- and middle-income countries
Source: Epidemics. 2024 Mar;46:100749. doi: 10.1016/j.epidem.2024.100749 (PMC10944168; doi:10.1016/j.epidem.2024.100749)
Supplement: Supplementary file 1 — Supplementary material [file mmc1.docx]

**Online Appendix for “Acquisition and Clearance Dynamics of *Campylobacter* spp. in Children in Low- and Middle-Income Countries”**

1. **Maximum-likelihood inference**

Despite the model being described in the form of a single-day transition, the time intervals of most consecutive pairs of the observed colonization events were more than one day given the sampling schedule of MAL-ED (1–3). As $p_{t}$and$q_{t}$ were allowed to vary from day to day, to simplify computation, we introduced average acquisition and clearance probabilities, respectively denoted by $\bar{p}_{il}$ and $\bar{q}_{il}$, for a multiple-day transition during the $l^{th}$ interval with a duration of $d_{il}$ days for person $i$ such that the transition matrix over the interval can be approximated by:

$$P_{il}=\left( \begin{matrix} 1-\bar{p}_{il} & \bar{p}_{il} \\ \bar{q}_{il} & 1-\bar{q}_{il} \end{matrix} \right)^{d_{il}}$$

We use approximation $\bar{p}_{il}\approx$($p_{t_{i(l-1)}}+p_{t_{i(l-0.5)}}+p_{t_{il}}$)/3 and $\bar{q}_{il}\approx\text{(}q_{t_{i\left( l-1 \right)}}+q_{t_{i(l-0.5)}}+q_{t_{il}}\text{)/3, }$where$\text{ }t_{i(l-1)}$, $t_{i(l-0.5)}$ and $t_{il}$ are the days at the beginning, median, and end of interval $l$ of person $i$. For interval $l$ofperson$i$, let $p_{il}(j,k)$ be the element of $P_{il}$ on row $j$ and column $k$ and $s_{i(l-1)}$ and $s_{il}$ be the infection states at the beginning and end, under the assumption that the colonization processes among children are independent, the likelihood of all data is:

$$L\left( \bar{p}_{il},\bar{q}_{il};y \right)=\prod_{i=1}^{N} \prod_{l=1}^{m_{i}} p_{il}(s_{i(l-1)},s_{il})$$

Where $m_{i}$ denotes the total number of multiple-day transitions of person $i: l\in\left( 1,..,m_{i} \right); N$ denotes the total number of children in a site: $i\in\left( 1,..,N \right)$. We assumed perfect sensitivity and specificity for the test assays (EIA and PCR) of the two *Campylobacter* groups.

We used maximum likelihood estimation (MLE) for statistical inference. We applied the BFGS algorithm to search the optimal log-likelihood for the parameters through the “optim” function in R (version 4.1.0), which was also used to perform other analyses. Based on the covariance matrix of the fundamental parameters, we applied the delta method to calculate the asymptotic 95% confidence intervals (CIs) of $p_{t}$ and $q_{t}$. The 95% CIs of $\frac{1}{p_{t}}$ and $\frac{1}{q_{t}}$ and FOI were calculated likewise. For both the MLE and the following model evaluation, we assumed all observations were absent from infection and diarrhea at birth (i.e., zero day of age).

1. **Model identifiability and selection**

In preliminary analyses of *Campylobacter* spp., we observed model identifiability issues at a couple of study sites. To illustrate, we show the estimates of $p_{t}$, $q_{t}$ and $p_{t}+q_{t}$ based on the 6-parameter full model ( $u_{t}=a+bt+ct^{2}$, $v_{t}=d+et+ft^{2}$) and the 5-parameter reduced model ( $u_{t}=a+bt$, $v_{t}=d+et+ft^{2}$) for *Campylobacter* spp. in Bangladesh and South Africa in Figure S1. With the full model, $p_{t}+q_{t}$ attained the upper bound *K*=0.3 near the end of year 2 for both countries, which contributed to extremely narrow confidence bands of $p_{t}$ and $q_{t}$ for those months. We deem the narrow confidence bands as “abnormal” because the EIA test data for *Campylobacter* spp. were less frequent in year 2 (quarterly) than in year 1 (monthly). In contrast, no unusual patterns were spotted for the estimates based on reduced model in either country. In addition, for each model we calculated the ratio of the maximum standard deviation to the median standard deviation among all parameter estimates. A large ratio indicates unusually large uncertainty in one or two parameter and thus lack of identifiability. We obtained ratios 20.39 for South Africa and 12.43 for Bangladesh based on the full model. In contrast, the numbers were 1.88 for South Africa and 1.31 for Bangladesh based on the reduced model, further confirming the lack of identifiability of the full model for the two countries. For countries that showed reasonable results from the full model, i.e., $p_{t}+q_{t}$ not reaching the upper bound, this ratio is reasonably small. For example, the full model for Tanzania had a ratio of 1.75, suggesting the model is well identified. In summary, we consider a model as non-identifiable if (1) the fitted curve of $p_{t}+q_{t}$ reaches its upper bound *K*, or mathematically, $\min_{t}\left( 1-\frac{p_{t}+q_{t}}{K} \right)$ is close to 0; or (2) the ratio $\frac{max(\mathrm{sd})}{\text{median}(\mathrm{sd})}$ is too large, where max(sd) and median(sd) denote the maximum and median of the standard deviations of the parameter estimates, respectively. Empirically, $\min_{t}\left( 1-\frac{p_{t}+q_{t}}{K} \right)<0.001$ and $\frac{max(\mathrm{sd})}{\text{median}(\mathrm{sd})}>10$ flag a potential issue of non-identifiability. As we have seen, these two issues tend to occur together. When a model is considered non-identifiable, we simplify the model by removing the term with the highest polynomial degree.

When multiple models are identifiable, we further use the Bayesian Information Criterion (BIC) and the determinant of the Hessian matrix to help with model selection, the latter being related to D-optimality in experimental design theory (4). A smaller BIC and a larger Det(Hessian) are preferred. In Table S1, we used the four criteria to perform a systematic comparison among the 4-, 5- and 6-parameter models for EIA-detected *Campylobacter* spp. in South Africa and Bangladesh and for PCR-detected *Campylobacter jejuli/coli* in South Africa. For *Campylobacter* spp. in South Africa and Bangladesh, $\min_{t}\left( 1-\frac{p_{t}+q_{t}}{K} \right)$ and $\frac{max(\mathrm{sd})}{\text{median}(\mathrm{sd})}$ clearly suggest the 6-parameter models are not identifiable, and both BIC and Det(Hessian) point to the 5-parameter models as the best choice at the two sites. For *C. jejuli/coli* in South Africa, all three models seem identifiable, but BIC and Det(Hessian) pick the 4-parameter model as the winner.

Based on these criteria, the final models chosen for each site and *Campylobacter* group are given in Tables S2 and S3. For the *Campylobacter* spp*.* data, we simplified the regression of $u_{t}$ for Bangladesh and South Africa. For the *Campylobacter* *jejuni/coli* data, we simplified $u_{t}$ for Brazil, Pakistan, Peru, and Tanzania, and we simplified both $u_{t}$ and $v_{t}$ for South Africa. For the *C. jejuni/coli* data from Brazil, because the observed prevalence was zero in the first two months of life (Figure 4 in the main text), we started model fitting from month two to improve identifiability. For the sensitivity analyses restricted to non-diarrheal samples, we used the same models chosen based on all samples.

To further verify estimability of the model parameters, we simulated 100 datasets of *Campylobacter* spp. at selected sites (Venda in South Africa and Haydom in Tanzania with low and high prevalence, respectively) using identifiable models estimated from real data and then estimated parameters for each simulated dataset. We examined both bias and mean square error (MSE) for the coefficients ($a, b, c, d, e, f$) in expression (1). Overall, parameters were identified accurately with small bias and variance when presence data are abundant, e.g., when the true parameters were based on the Tanzania data where prevalence was high (Table S4). When using parameters estimated from the South African data where prevalence was low, only one parameter ($a$) showed moderate bias, and all parameters had larger MSE than the Tanzania scenario. The median of the estimates for $a$ is however close to the true value. Overall, the proposed model is identifiable with adequate precision.

1. **Model evaluation**

Based on the estimated fundamental parameters ($a,b,c,d,e,f$) and their covariance matrix specific to each site and each *Campylobacter* group, we resampled 100 sets of the fundamental parameters and simulated epidemics for each setting without considering antibiotic use. The epidemics were simulated by sampling the colonization state on the later day of each single-day transition using a multinomial distribution conditioning on the colonization state on the corresponding earlier day of each transition. The simulated prevalence at the end of each month over the 24-month follow-up period was saved to represent the longitudinal trajectory. Goodness-of-fit of the model was assessed by comparing the mean curve of the 100 simulated trajectories of prevalence to the observed prevalence values. As the number of diarrheal samples was non-trivial (5), we fitted models and assessed goodness-of-fit for both all samples (diarrhea-triggered samples and routine surveillance samples) and routine surveillance samples only to investigate if diarrhea potentially introduced bias to the estimates.

1. **Characterizing effects of recent antibiotic use on clearance**

To study the association between recent antibiotic use and the daily clearance probability, we assume the antibiotic use affects $q_{t}$ in the following way:
$q_{t}=\left[ K*expit\left( u_{t} \right)*\left( 1-expit\left( v_{t} \right) \right) \right]^{\exp\left( \beta_{ML}*x_{ML}(t)+\beta_{FQ}*x_{FQ}(t)+\beta_{D}*x_{D}(t) \right)}$ (1)

$x_{ML}(t)$, $x_{FQ}(t)$, and $x_{D}(t)$ were respectively covariates for the use of macrolides (MLs), the use of fluoroquinolones (FQs), and diarrheal status during an n-day window preceding and including day t. Expression (1) is equivalent to a complementary log-log regression, as it can be re-written as

$$\log\left( -log\left( q_{t} \right) \right)=log\left( -log\left( K*expit\left( u_{t} \right)*\left( 1-expit\left( v_{t} \right) \right) \right) \right)+\beta_{ML}*x_{ML}\left( t \right)+\beta_{FQ}*x_{FQ}\left( t \right)+\beta_{D}*x_{D}\left( t \right).$$

We chose this parameterization instead of, say, logistic regression, because it is associated with stabler optimization of the likelihood. We considered diarrhea as a confounder, as diarrhea can lead to or be affected by antibiotic treatments (6), and diarrhea is a host response to enteric infection of pathogens. Determination of $x_{ML}(t), x_{FQ}(t), and x_{D}(t) is$ detailed as follows. If ≥ 1 course(s) of an antibiotic class or ≥ 1 time(s) of diarrhea were initiated during the n-day window prior to and including the day of a sampling event, the covariates = 1; otherwise, the covariates = 0. As antibiotics’ effect is usually short-term after administration, we set $n=8$, assuming the effect lasted about a week post-use. We conducted a sensitivity analysis for a sixteen-day window, and the results were generally similar to those of the eight-day window, with exceptions that additional significant effects of macrolides at the Nepal and Pakistan sites and of fluoroquinolones at the Pakistan site were observed, while the effects of fluoroquinolones at the Bangladesh site were no longer significant under the wider window (Figure S4).

For both the eight-day and sixteen-day windows, we did not report antibiotic effects at the South Africa and Brazil sites, where the frequencies of using macrolides and fluoroquinolones were too low to make $\beta_{ML}$and$\beta_{FQ}$ identifiable. At the Tanzania site, as the frequencies of fluoroquinolones for C. *jejuni/coli* were too low to make $\beta_{FQ}$ identifiable under both windows, we dropped $x_{FQ}(t)$ from the corresponding models. At the Nepal site under the eight-day window, few macrolides’ courses were associated with samples positive for C. *jejuni/coli* and *Campylobacter* spp., so we dropped $x_{ML}(t)$ and only evaluated $\beta_{FQ}$ and $\beta_{D}$ in the analysis for the two *Campylobacter* groups. At the Nepal site under the sixteen-day window, given the frequency of using macrolides for C. *jejuni/coli* was too low to yield identifiable $\beta_{ML}$, we only evaluated $\beta_{FQ}$ and $\beta_{D}$ for this scenario.

We present the antibiotic effect in the form of $e^{\beta}=\frac{\text{-}\text{log}q_{t}\left( x\left( t \right)=1 \right)}{\text{-}\text{log}q_{t}\left( x\left( t \right)=0 \right)}=\frac{\text{log}\left( 1/{q_{t}\left( x\left( t \right)=1 \right)} \right)}{\text{log}\left( 1/{q_{t}\left( x\left( t \right)=0 \right)} \right)}$, where $\beta$ can be $\beta_{ML}$ or $\beta_{FQ}$, $x\left( t \right)$ can be $x_{ML}(t)$ or $x_{FQ}(t)$, and$q_{t}\left( x\left( t \right)=1 \right)$ is the value of $q_{t}$ when $x\left( t \right)=1$. In other words, $e^{\beta}$ measures the ratio of the log durations of colonization with vs. without antibiotic use. An effective antibiotic corresponds to an increased clearance probability (i.e., $\frac{q_{t}\left( x\left( t \right)=1 \right)}{q_{t}\left( x\left( t \right)=0 \right)}>1$), and thus a shortened duration of colonization ($\frac{1/{q_{t}\left( x\left( t \right)=1 \right)}}{1/{q_{t}\left( x\left( t \right)=0 \right)}}<1$), i.e., $e^{\beta}<1$, which is analogous to a smaller relative risk of infection. We name $e^{\beta}$ the relative log time to clearance (RLTC). For example, when $e^{\beta}=0.5$, 1/$q_{t}\left( x\left( t \right)=1 \right)$ is the square root of ${1/q}_{t}\left( x\left( t \right)=0 \right)$. There is no one-to-one mapping between the RLTC and other measures such as relative risk and odds ratio. The relative risk and odds ratio depend not only on $e^{\beta}$ but also on the baseline value$q_{t}\left( x\left( t \right)=0 \right)$. To help readers with interpretation, we plot the ratio of times to clearance with versus without antibiotics,$\frac{1/{q_{t}\left( x\left( t \right)=1 \right)}}{1/{q_{t}\left( x\left( t \right)=0 \right)}}$, as a function of RLTC at different levels of$q_{t}\left( x\left( t \right)=0 \right)$ in Figure S5. When $q_{t}\left( x\left( t \right)=0 \right)=0.2$, $0.5\leq e^{\beta}\leq1$is mapped to $0.45\leq\frac{1/{q_{t}\left( x\left( t \right)=1 \right)}}{1/{q_{t}\left( x\left( t \right)=0 \right)}}\leq1$. When $q_{t}\left( x\left( t \right)=0 \right)=0.04$, $0.5\leq e^{\beta}\leq1$ is mapped to $0.2\leq\frac{1/{q_{t}\left( x\left( t \right)=1 \right)}}{1/{q_{t}\left( x\left( t \right)=0 \right)}}\leq1$.

**Table S1** Model identifiability and selection criteria for selected study sites (SAV:Venda, South Africa, BGD: Dhaka, Bangladesh) and models as examples. Models are indicated by the regression coefficient for $u_{t}$ and $v_{t}$. The arrow ↑ (↓) indicates a larger (smaller) value is desired. The models chosen as the final analysis are marked by *.

| Sites^*^ | Outcome | Model | $\min_{t}\left( 1-\frac{p_{t}+q_{t}}{K} \right)$ ↑ | $\frac{max(\mathrm{sd})}{\text{median}(\mathrm{sd})}$ ↓ | BIC ↓ | Det(Hessian) ↑ |
| --- | --- | --- | --- | --- | --- | --- |
| SAV | *Campylobacter* spp. | ($a$, $b$, $d$, $e$) | 0.136 | 5.431 | 2978 | 175499 |
|  |  | ($a$, $b$, $d$, $e,$ $f$)^*^ | 0.342 | 1.878 | 2974 | 264023 |
|  |  | ($a$, $b$, $c$, $d$, $e,$ $f$) | 1.09$\times{10}^{-4}$ | 20.391 | 2981 | 451 |
| BGD | *Campylobacter* spp. | ($a$, $b$, $d$, $e$) | 0.213 | 3.391 | 5104 | 13086021 |
|  |  | ($a$, $b$, $d$, $e,$ $f$)^*^ | 0.391 | 1.312 | 5071 | 52732040 |
|  |  | ($a$, $b$, $c$, $d$, $e,$ $f$) | 2.04$\times{10}^{-4}$ | 12.426 | 5077 | 386993 |
| SAV | *Campylobacter* *jejuni/coli* | ($a$, $b$, $d$, $e$)^*^ | 0.0138 | 6.0 | 1924 | 14330 |
|  |  | ($a$, $b$, $d$, $e,$ $f$) | 0.0251 | 2.148 | 1932 | 10983 |
|  |  | ($a$, $b$, $c$, $d$, $e,$ $f$) | 0.438 | 2.863 | 1938 | 14149 |

**Table S2** Fundamental parameters a, b, c estimated from full and non-diarrheal *Campylobacter* spp. and *Campylobacter* *jejuni/coli* data.

| Sites^*^ | *Campylobacter*  Groups | Samples | Regression Coefficients (95% CI) | | |
| --- | --- | --- | --- | --- | --- |
|  |  |  | $a$ | $b$ | $c$ |
| BGD | *C.* *jejuni/coli* | All | -2.93 (-3.46, -2.41) | 7.62 (5.13, 10.11) | -6.38 (-8.61, -4.15) |
|  |  | Non-diarrheal | -2.59 (-3.11, -2.07) | 3.55 (1.42, 5.67) | -2.90 (-4.77, -1.03) |
|  | *Campylobacter* spp. | All | -2.48 (-2.82, -2.15) | 2.85 (1.83, 3.86) | -^&^ |
|  |  | Non-diarrheal | -2.62 (-3.02, -2.23) | 1.85 (0.61, 3.09) | -^&^ |
| BRF | *C. jejuni/coli* | All | -1.09 (-2.22, 0.05) | -0.08 (-1.93, 1.77) | -^&^ |
|  |  | Non-diarrheal | -1.30 (-2.42, -0.19) | 0.10 (-1.69, 1.90) | -^&^ |
|  | *Campylobacter* spp. | All | -2.53 (-3.81, -1.25) | 4.43 (-1.02, 9.89) | -4.57 (-9.22, 0.08) |
|  |  | Non-diarrheal | -2.20 (-3.58, -0.81) | 2.95 (-2.94, 8.84) | -3.71 (-8.80, 1.37) |
| INV | *C.* *jejuni/coli* | All | -2.52 (-3.20, -1.83) | 4.18 (1.25, 7.12) | -3.07 (-5.63, -0.51) |
|  |  | Non-diarrheal | -2.34 (-3.06, -1.62) | 2.74 (-0.23, 5.72) | -1.96 (-4.54, 0.61) |
|  | *Campylobacter* spp. | All | -2.44 (-2.95, -1.93) | 3.48 (1.21, 5.75) | -3.94 (-5.97, -1.90) |
|  |  | Non-diarrheal | -2.71 (-3.25, -2.17) | 3.56 (1.22, 5.89) | -4.37 (-6.42, -2.33) |
| NEB | *C.* *jejuni/coli* | All | -3.74 (-4.46, -3.02) | 8.00 (5.06, 10.94) | -5.25 (-7.76, -2.75) |
|  |  | Non-diarrheal | -3.32 (-4.09, -2.56) | 5.79 (2.77, 8.81) | -3.64 (-6.18, -1.11) |
|  | *Campylobacter* spp. | All | -2.21 (-2.75, -1.67) | 3.80 (1.12, 6.48) | -2.83 (-5.69, 0.02) |
|  |  | Non-diarrheal | -2.48 (-3.05, -1.92) | 4.79 (2.28, 7.30) | -5.55 (-7.80, -3.30) |
| PEL | *C.* *jejuni/coli* | All | -0.95 (-1.57, -0.33) | 0.64 (-0.48, 1.75) | -^&^ |
|  |  | Non-diarrheal | -1.75 (-2.19, -1.31) | 0.60 (-0.15, 1.35) | -^&^ |
|  | *Campylobacter* spp. | All | -0.97 (-1.69, -0.25) | -0.53 (-3.35, 2.29) | -0.24 (-2.72, 2.24) |
|  |  | Non-diarrheal | -1.84 (-2.47, -1.21) | 0.58 (-2.09, 3.24) | -1.71 (-4.09, 0.66) |
| PKN | *C.* *jejuni/coli* | All | -0.37 (-0.94, 0.20) | -0.83 (-1.67, 0.01) | -^&^ |
|  |  | Non-diarrheal | -0.86 (-1.52, -0.20) | -0.66 (-1.58, 0.26) | -^&^ |
|  | *Campylobacter* spp. | All | -1.95 (-2.28, -1.62) | 1.20 (-0.64, 3.05) | -0.31 (-2.51, 1.89) |
|  |  | Non-diarrheal | -2.41 (-2.79, -2.04) | 2.80 (0.97, 4.62) | -3.67 (-5.39, -1.94) |
| SAV | *C.* *jejuni/coli* | All | -2.63 (-3.52, -1.73) | 6.75 (2.38, 11.13) | -^&^ |
|  |  | Non-diarrheal | -2.38 (-3.46, -1.30) | 5.36 (-0.02, 10.74) | -^&^ |
|  | *Campylobacter* spp. | All | -1.49 (-2.26, -0.73) | 2.11 (-0.73, 4.94) | -^&^ |
|  |  | Non-diarrheal | -1.50 (-2.18, -0.81) | 1.59 (-0.73, 3.91) | -^&^ |
| TZH | *C.* *jejuni/coli* | All | -1.56 (-1.83, -1.29) | -0.71 (-1.13, -0.29) | -^&^ |
|  |  | Non-diarrheal | -1.64 (-1.92, -1.36) | -0.60 (-1.03, -0.17) | -^&^ |
|  | *Campylobacter* spp. | All | -1.95 (-2.55, -1.35) | 2.00 (-0.68, 4.67) | -3.18 (-5.52, -0.84) |
|  |  | Non-diarrheal | -1.95 (-2.57, -1.32) | 1.59 (-1.16, 4.33) | -2.94 (-5.31, -0.56) |

^*^Sites included: Dahka, Bangladesh (BGD); Vallore, India (INV); Bhaktapur, Nepal (NEB); Naushero Feroze, Pakistan (PKN); Venda, South Africa (SAV); Haydom, Tanzania (TZH); Fortaleza, Brazil (BRF); Loreto, Peru (PEL).

^&^Given preliminary analysis indicated an identifiability issue existed when parameter *c* was kept in the model for this setting, this parameter was excluded in formal analysis.

**Table S3** Fundamental parameters d, e, f estimated from full and non-diarrheal *Campylobacter* spp. and *Campylobacter* *jejuni/coli* data.

| Sites^*^ | *Campylobacter* groups | Samples | Regression Coefficients (95% CI) | | |
| --- | --- | --- | --- | --- | --- |
|  |  |  | $d$ | $e$ | $f$ |
| BGD | *C.* *jejuni/coli* | All | -2.13 (-2.49, -1.76) | 4.86 (3.47, 6.24) | -3.57 (-4.77, -2.37) |
|  |  | Non-diarrheal | -2.20 (-2.60, -1.79) | 4.87 (3.32, 6.42) | -3.50 (-4.86, -2.14) |
|  | *Campylobacter* spp. | All | -1.89 (-2.22, -1.56) | 6.87 (5.53, 8.20) | -4.08 (-5.28, -2.89) |
|  |  | Non-diarrheal | -1.77 (-2.16, -1.38) | 6.36 (4.71, 8.01) | -3.77 (-5.27, -2.27) |
| BRF | *C. jejuni/coli* | All, | -3.73 (-4.67, -2.79) | 4.20 (0.42, 7.98) | -4.34 (-7.77, -0.92) |
|  |  | Non-diarrheal | -4.00 (-5.04, -2.97) | 5.26 (1.12, 9.41) | -5.42 (-9.19, -1.65) |
|  | *Campylobacter* spp. | All | -3.58 (-4.39, -2.76) | 7.03 (3.52, 10.53) | -7.09 (-10.50, -3.68) |
|  |  | Non-diarrheal | -3.91 (-4.74, -3.07) | 8.78 (5.01, 12.54) | -9.26 (-13.15, -5.36) |
| INV | *C.* *jejuni/coli* | All | -2.24 (-2.69, -1.78) | 3.36 (1.53, 5.18) | -4.02 (-5.68, -2.36) |
|  |  | Non-diarrheal | -2.34 (-2.81, -1.88) | 3.69 (1.79, 5.58) | -4.27 (-6.01, -2.53) |
|  | *Campylobacter* spp. | All | -2.02 (-2.40, -1.64) | 5.19 (3.58, 6.80) | -4.21 (-5.71, -2.71) |
|  |  | Non-diarrheal | -1.97 (-2.41, -1.53) | 5.23 (3.34, 7.12) | -4.37 (-6.15, -2.58) |
| NEB | *C.* *jejuni/coli* | All | -2.26 (-2.83, -1.70) | 5.09 (3.10, 7.08) | -5.05 (-6.68, -3.42) |
|  |  | Non-diarrheal | -2.44 (-3.01, -1.86) | 5.53 (3.48, 7.58) | -5.35 (-7.05, -3.66) |
|  | *Campylobacter* spp. | All | -2.06 (-2.41, -1.71) | 3.28 (1.82, 4.73) | -2.34 (-3.67, -1.02) |
|  |  | Non-diarrheal | -2.03 (-2.43, -1.63) | 3.16 (1.42, 4.91) | -2.36 (-4.02, -0.70) |
| PEL | *C.* *jejuni/coli* | All | -3.09 (-3.42, -2.76) | 7.55 (6.22, 8.88) | -6.81 (-8.01, -5.60) |
|  |  | Non-diarrheal | -3.26 (-3.69, -2.83) | 7.74 (6.07, 9.42) | -6.83 (-8.33, -5.34) |
|  | *Campylobacter* spp. | All | -2.59 (-2.89, -2.29) | 6.14 (4.84, 7.44) | -4.59 (-5.81, -3.37) |
|  |  | Non-diarrheal | -2.69 (-3.10, -2.27) | 6.64 (4.82, 8.47) | -5.17 (-6.92, -3.43) |
| PKN | *C.* *jejuni/coli* | All | -3.00 (-3.28, -2.71) | 6.53 (5.33, 7.72) | -5.84 (-6.97, -4.72) |
|  |  | Non-diarrheal | -2.99 (-3.33, -2.65) | 6.54 (5.11, 7.96) | -5.96 (-7.29, -4.62) |
|  | *Campylobacter* spp. | All | -1.11 (-1.35, -0.87) | 5.98 (4.90, 7.05) | -5.38 (-6.39, -4.37) |
|  |  | Non-diarrheal | -1.03 (-1.34, -0.71) | 6.10 (4.65, 7.55) | -5.81 (-7.26, -4.36) |
| SAV | *C.* *jejuni/coli* | All | -2.10 (-2.41, -1.79) | -1.49 (-2.05, -0.93) | -^&^ |
|  |  | Non-diarrheal | -2.19 (-2.51, -1.87) | -1.45 (-2.03, -0.86) | -^&^ |
|  | *Campylobacter* spp. | All | -2.28 (-2.61, -1.95) | 2.65 (1.12, 4.17) | -2.67 (-4.18, -1.16) |
|  |  | Non-diarrheal | -2.31 (-2.64, -1.97) | 2.59 (1.02, 4.16) | -2.62 (-4.18, -1.07) |
| TZH | *C.* *jejuni/coli* | All | -1.75 (-2.03, -1.47) | 5.52 (4.27, 6.76) | -4.73 (-5.92, -3.54) |
|  |  | Non-diarrheal | -1.75 (-2.04, -1.46) | 5.49 (4.22, 6.76) | -4.69 (-5.89, -3.49) |
|  | *Campylobacter* spp. | All | -2.18 (-2.53, -1.83) | 7.80 (6.24, 9.37) | -6.07 (-7.57, -4.58) |
|  |  | Non-diarrheal | -2.14 (-2.50, -1.78) | 7.66 (6.04, 9.27) | -5.99 (-7.54, -4.44) |

^*^Sites included: Dahka, Bangladesh (BGD); Vallore, India (INV); Bhaktapur, Nepal (NEB); Naushero Feroze, Pakistan (PKN); Venda, South Africa (SAV); Haydom, Tanzania (TZH); Fortaleza, Brazil (BRF); Loreto, Peru (PEL).

^&^Given preliminary analysis indicated an identifiability issue existed when parameter *f* was kept in the model for this setting, this parameter was excluded in formal analysis.

**Table S4** Model validation: median, inter-quartile range (IQR), mean and mean square error (MSE) of estimated model coefficients ($a$, $b$, $c$, $d$, $e,$ and $f$) over 100 simulated datasets. Data-generating parameters were estimated from the *Campylobacter* spp. data from sites in South Africa and Tanzania.

| Sites^*^ |  | *a* | *b* | *c* | *d* | *e* | *f* |
| --- | --- | --- | --- | --- | --- | --- | --- |
| SAV | True parameters  (95% CI)^^^ | -1.49  (-2.26, -0.73) | 2.11  (-0.73, 4.94) | -^&^ | -2.28  (-2.61, -1.95) | 2.65  (1.12, 4.17) | -2.67  (-4.18, -1.16) |
|  | Median (IQR) of estimates^^*^ | -1.52 (0.77) | 2.17 (3.28) | -^&^ | -2.29 (0.29) | 2.66 (1.30) | -2.67 (1.18) |
|  | Mean (MSE) of estimates^^*^ | -1.14 (5.38) | 2.25 (9.05) | -^&^ | -2.27 (0.02) | 2.63 (0.44) | -2.68 (0.39) |
| TZH | True parameters  (95% CI)^^^ | -1.95  (-2.55, -1.35) | 2.00  (-0.68, 4.67) | -3.18  (-5.52, -0.84) | -2.18  (-2.53, -1.83) | 7.80  (6.24, 9.37) | -6.07  (-7.57, -4.58) |
|  | Median (IQR) of estimates^^*^ | -1.94 (0.61) | 2.01 (2.39) | -3.15 (1.93) | -2.21 (0.33) | 7.94 (1.51) | -6.16 (1.43) |
|  | Mean (MSE) of estimates^^*^ | -1.93 (0.05) | 1.95 (0.77) | -3.12 (0.60) | -2.18 (0.03) | 7.91 (0.53) | -6.21 (0.53) |

^&^Given preliminary analysis indicated an identifiability issue existed in the model when parameter *c* was kept in the model for the data at this site, this parameter was excluded for formal analysis.

^^^Parameters estimated from the observed data were described as true parameters in the simulation study. With the true parameters, we simulated 100 epidemics of *Campylobacter* spp. infection for each site. We fitted the Markov model to these 100 epidemics to estimate the parameters for each epidemic, described as estimated parameters, and reported their medians and means.

^*^SAV – Venda, South Africa; TZH – Haydom, Tanzania; IQR – interquartile range; MSE – mean squared error.

**Table S5** Model-estimated mean time to acquisition and mean time to clearance (or duration of colonization) of *Campylobacter* *jejuni/coli* and *Campylobacter* spp. in the first two years of age. These estimates were obtained by taking the median and average of the model-estimated real-time mean times to acquisition or clearance over the study period.

| Sites^*^ |  | Median  (95% CI) | Mean  (95% CI) |
| --- | --- | --- | --- |
|  | Time to acquisition (days) |  |  |
| BGD | *C. jejuni/coli* | 39 (33, 47) | 57 (50, 64) |
|  | *Campylobacter* spp. | 21 (17, 25) | 28 (24, 33) |
| BRF | *C. jejuni/coli* | 274 (177, 422) | 306 (215, 437) |
|  | *Campylobacter* spp. | 252 (137, 462) | 328 (229, 468) |
| INV | *C. jejuni/coli* | 125 (101, 155) | 143 (125, 164) |
|  | *Campylobacter* spp. | 88 (67, 114) | 100 (86, 116) |
| NEB | *C. jejuni/coli* | 70 (59, 83) | 104 (92, 118) |
|  | *Campylobacter* spp. | 54 (44, 66) | 68 (55, 84) |
| PEL | *C. jejuni/coli* | 49 (40, 61) | 60 (51, 71) |
|  | *Campylobacter* spp. | 57 (44, 74) | 64 (55, 75) |
| PKN | *C. jejuni/coli* | 62 (51, 74) | 72 (61, 84) |
|  | *Campylobacter* spp. | 30 (26, 35) | 35 (30, 42) |
| SAV | *C. jejuni/coli* | 108 (83, 140) | 132 (102, 170) |
|  | *Campylobacter* spp. | 55 (35, 86) | 67 (45, 99) |
| TZH | *C. jejuni/coli* | 70 (62, 79) | 75 (68, 83) |
|  | *Campylobacter* spp. | 62 (48, 79) | 74 (64, 86) |
|  | Time to clearance (days) |  |  |
| BGD | *C. jejuni/coli* | 20 (17, 23) | 23 (20, 26) |
|  | *Campylobacter* spp. | 35 (28, 43) | 33 (28, 39) |
| BRF | *C. jejuni/coli* | 14 (8, 25) | 14 (10, 20) |
|  | *Campylobacter* spp. | 25 (15, 44) | 28 (20, 38) |
| INV | *C. jejuni/coli* | 18 (12, 26) | 21 (18, 24) |
|  | *Campylobacter* spp. | 39 (31, 49) | 42 (37, 49) |
| NEB | *C. jejuni/coli* | 18 (15, 21) | 25 (21, 29) |
|  | *Campylobacter* spp. | 18 (10, 32) | 20 (16, 25) |
| PEL | *C. jejuni/coli* | 12 (9, 18) | 12 (10, 14) |
|  | *Campylobacter* spp. | 23 (20, 28) | 22 (19, 25) |
| PKN | *C. jejuni/coli* | 14 (12, 16) | 13 (11, 15) |
|  | *Campylobacter* spp. | 40 (34, 47) | 37 (32, 44) |
| SAV | *C. jejuni/coli* | 5 (3, 8) | 8 (6, 10) |
|  | *Campylobacter* spp. | 10 (6, 17) | 10 (7, 15) |
| TZH | *C. jejuni/coli* | 48 (42, 53) | 41 (37, 46) |
|  | *Campylobacter* spp. | 50 (40, 63) | 53 (45, 61) |

*Sites included: Dahka, Bangladesh (BGD); Vallore, India (INV); Bhaktapur, Nepal (NEB); Naushero Feroze, Pakistan (PKN); Venda, South Africa (SAV); Haydom, Tanzania (TZH); Fortaleza, Brazil (BRF); Loreto, Peru (PEL).


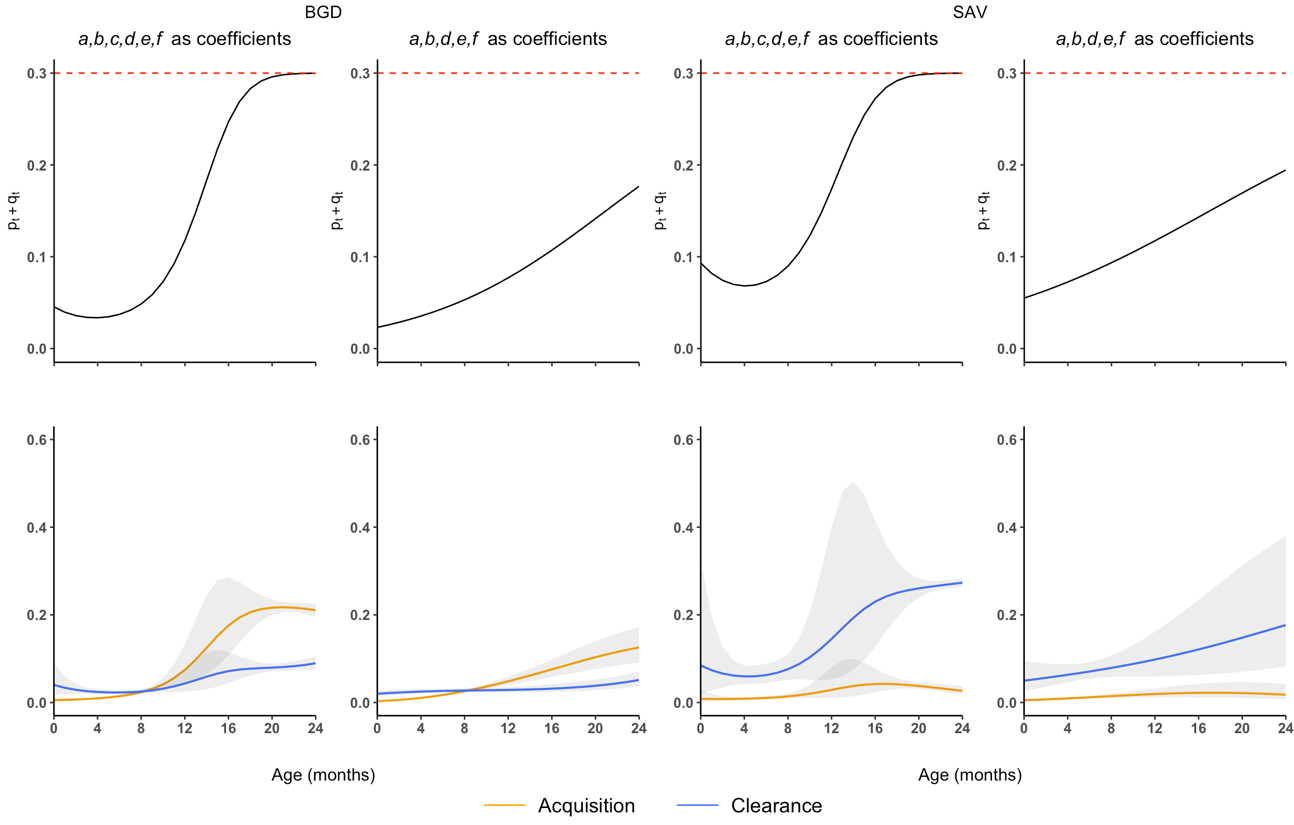


Figure S1 Model-estimated daily acquisition (orange, $\boldsymbol{p}_{\boldsymbol{t}}$) and clearance (blue, $\boldsymbol{q}_{\boldsymbol{t}}$) probabilities of EIA-detected Campylobacter spp. (lower panel) and their sums (upper panel) at sites of Dhaka, Bangladesh (BGD) and Venda, South Africa (SAV). 95% asymptotic confidence intervals are shown as gray error bands. The upper bound $\boldsymbol{K}$ of 0.3 is marked by a red dash line.


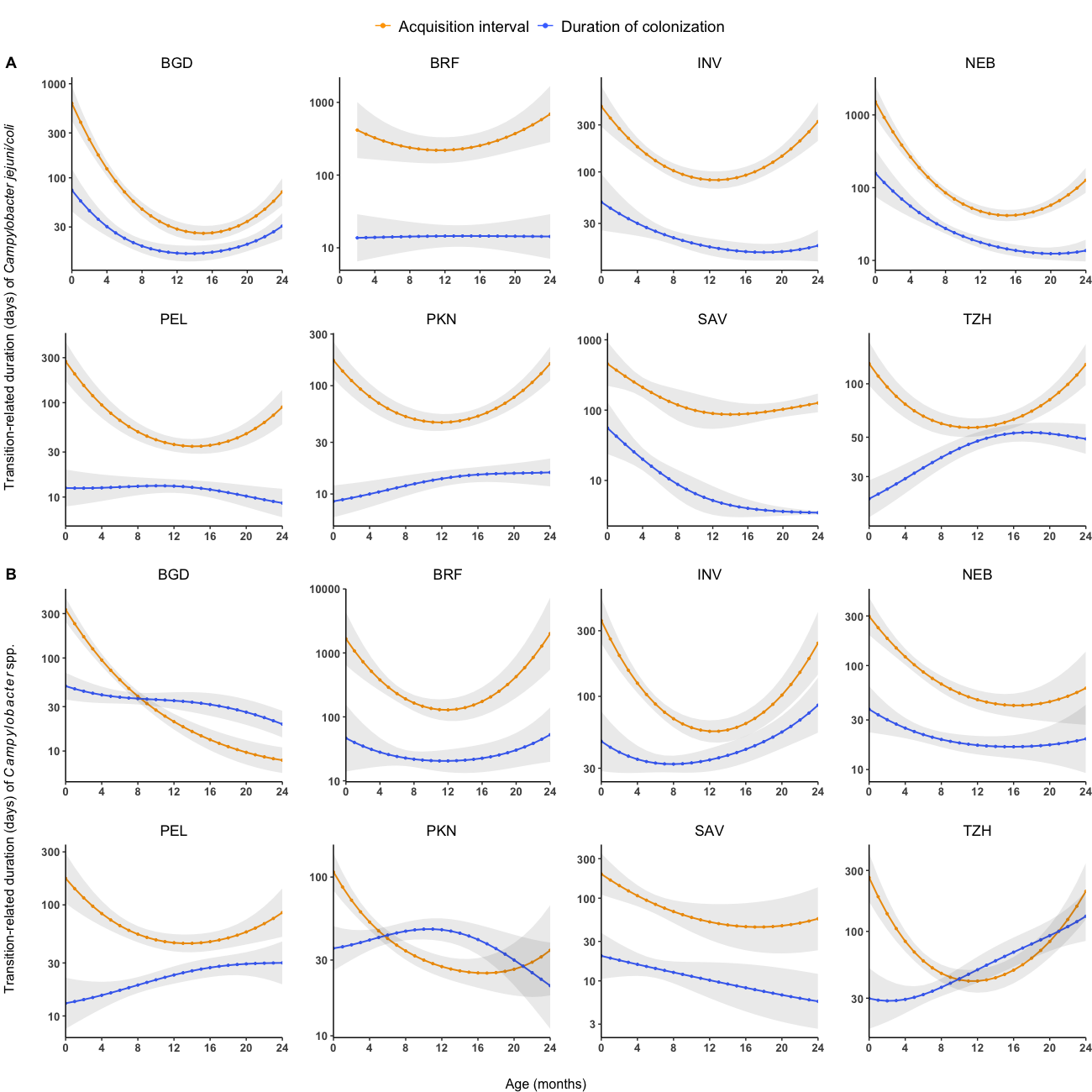


**Figure S2** Model-estimated site-specific age-dependent mean time to acquisition ($\frac{\boldsymbol{1}}{\boldsymbol{p}_{\boldsymbol{t}}}$, orange) and mean time to clearance ($\frac{\boldsymbol{1}}{\boldsymbol{q}_{\boldsymbol{t}}}$, blue) for *Campylobacter* *jejuni/coli* (A) and *Campylobacter* spp. (B). Grey shades are 95% asymptotic confidence bands. Sites included: Dhaka, Bangladesh (BGD); Vallore, India (INV); Bhaktapur, Nepal (NEB); Naushero Feroze, Pakistan (PKN); Venda, South Africa (SAV); Haydom, Tanzania (TZH); Fortaleza, Brazil (BRF); Loreto, Peru (PEL).


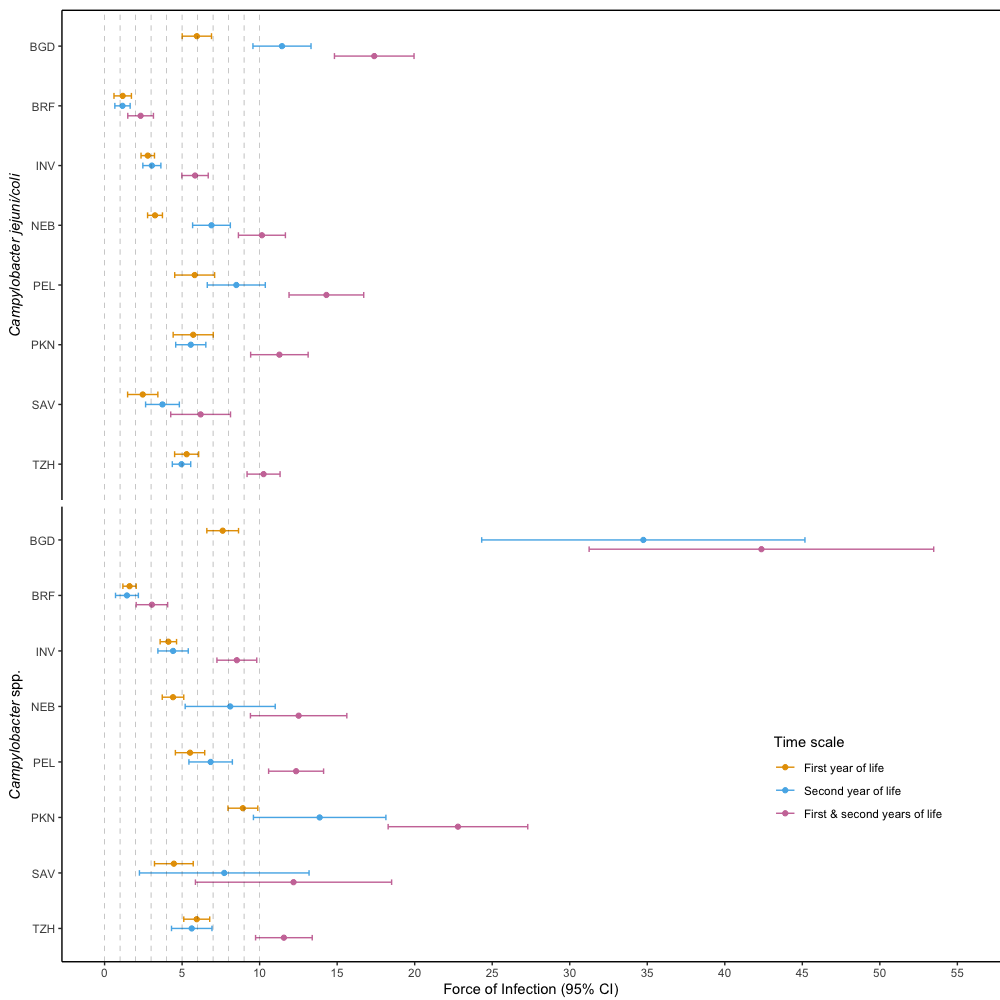


**Figure S3** Site-specific forces of infection of *Campylobacter* *jejuni/coli* and *Campylobacter* spp. Sites included: Dhaka, Bangladesh (BGD); Vallore, India (INV); Bhaktapur, Nepal (NEB); Naushero Feroze, Pakistan (PKN); Venda, South Africa (SAV); Haydom, Tanzania (TZH); Fortaleza, Brazil (BRF); Loreto, Peru (PEL).


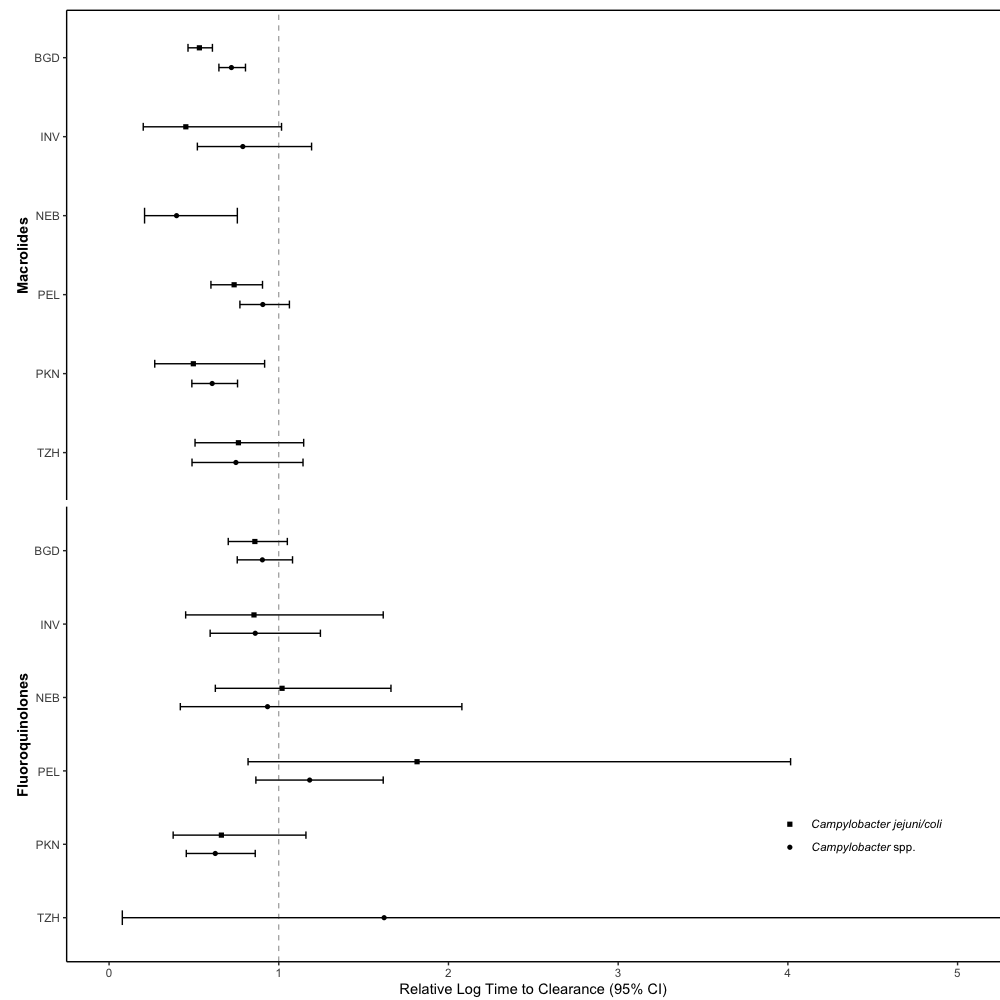


**Figure S4** Site-specific effects of the use of macrolides and fluoroquinolones during prior sixteen days on the clearances of *Campylobacter jejuni/coli* and *Campylobacter* spp. Point estimates and 95% confidence intervals of the relative log time to clearance (RLTC) were shown, which is defined as the ratio of the log time to clearance with antibiotic use to the log time to clearance without. The upper confidence interval limit (i.e., 33.33) of RLTC of fluoroquinolones on *Campylobacter* spp. in TZH was not shown. Sites included: Dhaka, Bangladesh (BGD); Vallore, India (INV); Bhaktapur, Nepal (NEB); Naushero Feroze, Pakistan (PKN); Venda, South Africa (SAV); Haydom, Tanzania (TZH); Fortaleza, Brazil (BRF); Loreto, Peru (PEL).


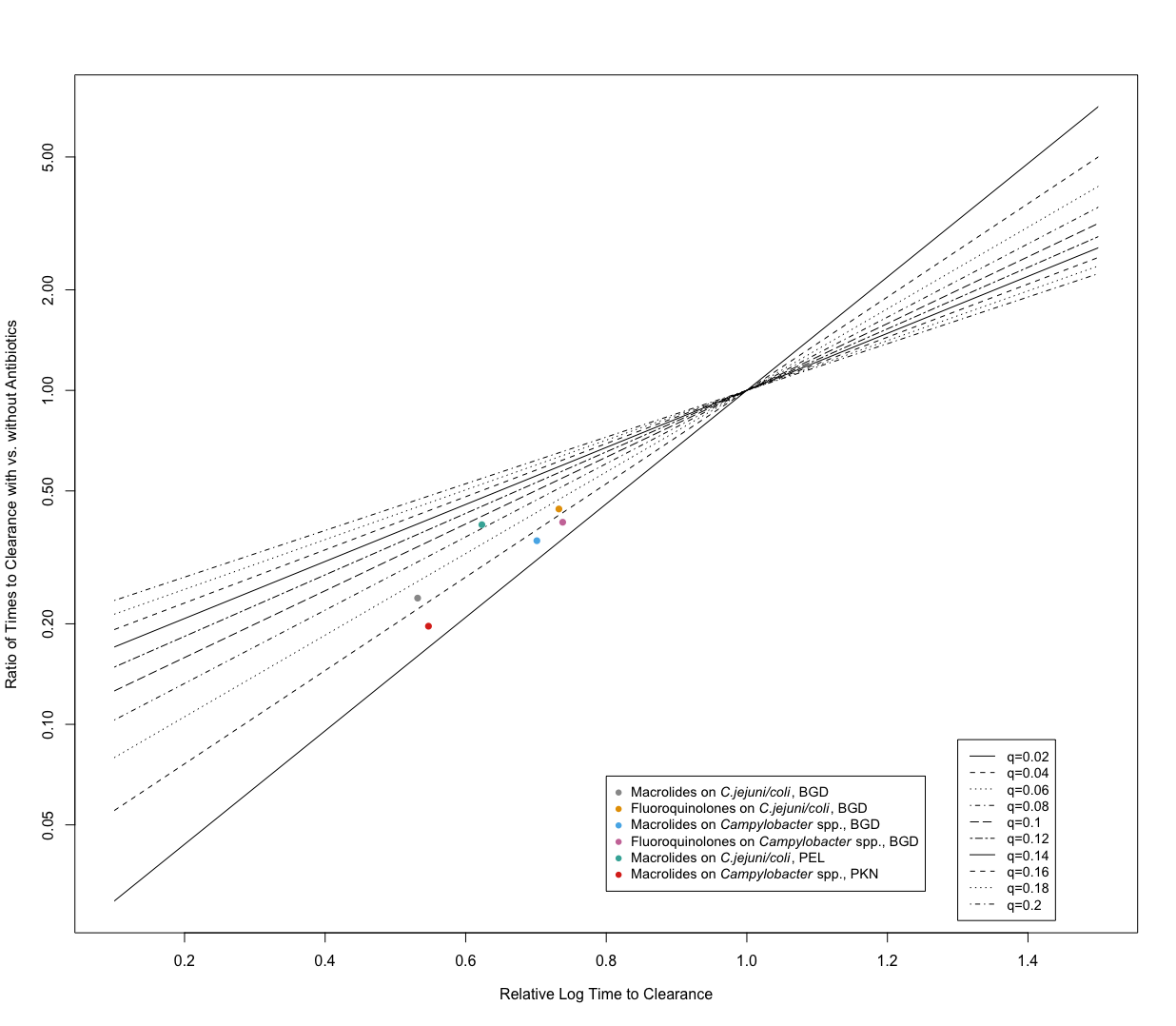


**Figure S5** Relationship between the ratio of times to clearance with vs. without antibiotics, ($\frac{1/{q_{t}\left( x\left( t \right)=1 \right)}}{1/{q_{t}\left( x\left( t \right)=0 \right)}})$ and the relative log time to clearance (RLTC) at various levels of baseline clearance probability$q_{t}\left( x\left( t \right)=0 \right)$. The Y-axis is at the log scale. We use RLTC as the main metric for the antibiotic effect on clearance of *Campylobacter* spp. The ratios of time to clearance corresponding to the statistically significant RLTCs in Dhaka, Bangladesh (BGD), Naushero Feroze, Pakistan (PKN) and Loreto, Peru (PEL) are marked by colored point. Calculation of the ratios was based on the mean daily clearance probability during the 24-month follow-up for each *Campylobacter* group at each site.

**Appendix References**

1. Miller M, Acosta AM, Chavez CB, Flores JT, Olotegui MP, Pinedo SR, et al. The MAL-ED study: a multinational and multidisciplinary approach to understand the relationship between enteric pathogens, malnutrition, gut physiology, physical growth, cognitive development, and immune responses in infants and children up to 2 years of age in resource-poor environments. Clin Infect Dis. 2014 Nov 1;59 Suppl 4:S193–206.

2. Amour C, Gratz J, Mduma ER, Svensen E, Rogawski ET, McGrath M, et al. Epidemiology and Impact of Campylobacter Infection in Children in 8 Low-Resource Settings: Results from the MAL-ED Study. Clinical Infectious Diseases. 2016;

3. Houpt E, Gratz J, Kosek M, Zaidi AKM, Qureshi S, Kang G, et al. Microbiologic methods utilized in the MAL-ED cohort study. Clin Infect Dis. 2014 Nov 1;59 Suppl 4(Suppl 4):S225–32.

4. Emery AF, Nenarokomov A V. Optimal experiment design. Meas Sci Technol [Internet]. 1998 Jun 1;9(6):864–76. Available from: https://iopscience.iop.org/article/10.1088/0957-0233/9/6/003

5. Rogawski ET, Liu J, Platts-Mills JA, Kabir F, Lertsethtakarn P, Siguas M, et al. Use of quantitative molecular diagnostic methods to investigate the effect of enteropathogen infections on linear growth in children in low-resource settings: longitudinal analysis of results from the MAL-ED cohort study. Lancet Global Health. 2018;6(12):E1319–28.

6. Rogawski ET, Platts-Mills JA, Seidman JC, John S, Mahfuz M, Ulak M, et al. Use of antibiotics in children younger than two years in eight countries: a prospective cohort study. Bull World Health Organ. 2017;
